# Dynamic applicability domain (dAD): compound–target binding affinity estimates with local conformal prediction

**DOI:** 10.1093/bioinformatics/btad465

**Published:** 2023-08-18

**Authors:** Davor Oršolić, Tomislav Šmuc

**Affiliations:** Division of Electronics, Ruđer Bošković Institute, Bijenička cesta 54, Zagreb 10000, Croatia; Division of Electronics, Ruđer Bošković Institute, Bijenička cesta 54, Zagreb 10000, Croatia

## Abstract

**Motivation:**

Increasing efforts are being made in the field of machine learning to advance the learning of robust and accurate models from experimentally measured data and enable more efficient drug discovery processes. The prediction of binding affinity is one of the most frequent tasks of compound bioactivity modelling. Learned models for binding affinity prediction are assessed by their average performance on unseen samples, but point predictions are typically not provided with a rigorous confidence assessment. Approaches, such as the conformal predictor framework equip conventional models with a more rigorous assessment of confidence for individual point predictions. In this article, we extend the inductive conformal prediction framework for interaction data, in particular the compound–target binding affinity prediction task. The new framework is based on dynamically defined calibration sets that are specific for each testing pair and provides prediction assessment in the context of calibration pairs from its compound–target neighbourhood, enabling improved estimates based on the local properties of the prediction model.

**Results:**

The effectiveness of the approach is benchmarked on several publicly available datasets and tested in realistic use-case scenarios with increasing levels of difficulty on a complex compound–target binding affinity space. We demonstrate that in such scenarios, novel approach combining applicability domain paradigm with conformal prediction framework, produces superior confidence assessment with valid and more informative prediction regions compared to other ‘state-of-the-art’ conformal prediction approaches.

**Availability and implementation:**

Dataset and the code are available on GitHub (https://github.com/mlkr-rbi/dAD).

## 1 Introduction

The fast growth of experimental results published in the scientific literature and through repositories makes modelling of binding affinity between compounds and protein targets expanding and interesting from both scientific and industrial aspects. Methods for *in silico* modelling and screening of large chemical compound spaces are often computational pipelines based on feature generation tools and machine-learning algorithms (Cichonska *et al.* 2017, Öztürk *et al.* 2018, Cichońska *et al.* 2021, Nguyen *et al.* 2021). Compound and target spaces can be described with a multitude of different descriptors, including those that describe their structural or physicochemical properties (Lim *et al.* 2021). In Öztürk *et al.* (2018), authors used convolutional neural networks to learn small molecule and protein representations from 1D sequences. On the other hand, to improve the predictive power of the model with a more realistic representation of molecules, the GraphDTA method was proposed in Nguyen *et al.* (2021). This approach is based on a graph convolutional block that learns compound representations from a molecular graph (Kipf and Welling 2016). In QSAR modelling, predictive models must not only aim for high accuracy on unseen samples, but they must also be accompanied by estimations of the prediction region with a certain degree of confidence. Conventional QSAR modelling uses applicability domain (AD) to improve prediction credibility (Gadaleta *et al.* 2016). AD of the QSAR model represents a bounded chemical space within which the model is guaranteed a well-defined and reliable performance on average (Aniceto *et al.* 2016, Mathea *et al.* 2016, Klingspohn *et al.* 2017). However, the concept of AD does not provide an apparatus that would determine how reliable certain model predictions are (Aniceto *et al.* 2016). When using AD, the user determines the portion of external data that falls within the established boundaries, without assessing the AD’s ability to differentiate between ‘acceptable’ and ‘unacceptable’ new predictions (Aniceto *et al.* 2016, Mathea *et al.* 2016). Intuitively, the AD increases the confidence of the model’s predictions, but this is not directly quantified. According to Aniceto *et al.* (2016), AD is set using training sample similarity thresholds or class probability estimates, etc. This approach treats AD as a space between the defined limits typically overlooking the possibility of localized holes in the chemical space where the model’s predictions may be unreliable (Klingspohn *et al.* 2017). Furthermore, a well-balanced AD formulation would need to include information on distribution of both entities when dealing with interacting pairs.

Conformal prediction (CP) framework was introduced by Gammerman *et al.* (1998), with the intention of providing confidence for classification predictions made by the support vector machines. First attempt at CP was made using transductive conformal predictors, which required retraining of the model for each individual prediction and were therefore computationally expensive (Shafer and Vovk 2008). As a result, the inductive conformal prediction (ICP) framework was developed (Papadopoulos 2008). The ICP framework uses a calibration set, that typically accounts for a smaller portion of the training samples, to calibrate the trained model and compute confidence levels. The disadvantage of taking such an approach for regression problems is that the prediction regions computed based on the calibration set for any particular level of confidence are fixed, meaning that they do not alter from test sample to test sample (Johansson *et al.* 2014). In the CP framework, AD related measures (Klingspohn *et al.* 2017) can also be used for conformity scoring to locate the AD or the calibration set, for each individual test sample. We call this set a conformity region of sample *x*.

The typical non-conformity function that is utilized for regression tasks is the absolute difference between the true label and the predicted label for a particular sample, $\left|y_{i}-\hat{y_{i}}\right|$ (Shafer and Vovk 2008, Papadopoulos and Haralambous 2010, Papadopoulos *et al.* 2011, Johansson *et al.* 2014). This approach focuses solely on the non-conformity in the label space, and assumes that training and calibration sets must be exchangeable (Shafer and Vovk 2008). For a predefined confidence level prediction regions are fixed, meaning they remain the same for any new test sample tested which prevents them from being as informative as we would like them to be. In order to alleviate this, later studies introduced normalization steps in the context of non-conformity function definition (Papadopoulos and Haralambous 2010, Papadopoulos *et al.* 2011).

In this article, we introduce the CP framework that is better suited for the interaction data character of the compound–target binding affinity modelling task. We rationalize that binding affinity modelling task, or for that matter, any dataset containing interactions between two entities, requires special treatment when defining non-conformity in the space of interacting pairs. For binding affinity modelling specifically, binding affinities are conditioned on input data that comes from two distinct distributions: one of compounds and one of targets. Conformal predictors based solely on predictions, i.e. distribution of errors in the label space, cannot reflect the true non-conformity of the sample in the space of predictor variables. We thus aim to expand the CP framework for this type of problem by introducing the concept of the dynamic calibration set, the calibration set that is specific for the particular compound–target pair being tested. The ‘localised’ calibration based on the dynamic calibration set should provide more precise non-conformity scores for tested samples and also better performance in cases when testing samples are found outside the boundaries of AD or out-of-distribution.

We test our method and compare it against other ‘state-of-the-art’ approaches over several benchmark compound–target binding affinity datasets, as well as a specifically designed small compound–protein kinase binding affinity dataset that is constructed to allow the testing of the conformal predictor frameworks in settings that are representative of the real use-case scenarios.

## 2 Proposed CP framework

The initial step in a conventional ICP framework is to divide the training set into a proper training set and a calibration set (Shafer and Vovk 2008, Alvarsson *et al.* 2021). The calibration set must reflect the distribution of the training samples, satisfying the assumption that the data are independent and identically distributed, or a more relaxed assumption that they are exchangeable (Shafer and Vovk 2008, Papadopoulos *et al.* 2011). Contrary to the conventional calibration set definition, which is stationary and represents the overall training space, we define a dynamic calibration set for each individual test sample by locating the most conforming samples of the training landscape to the sample that is being tested. Let us denote the training set of compound–target pairs as: where $x_{i}$ is a compound–target pair and $y_{i}$ is a measured binding affinity for that pair. Let the set of compounds and targets be denoted by *C* and *T*, respectively, and the corresponding set of training compound–target pairs by X = (C,T).


(1) $$Z=(x_{1},y_{1}),\ldots ,(x_{m},y_{m}),$$

Calibration set ($Z^{c}$) of a new test sample is dynamically constructed from training samples that have maximum Tanimoto similarity coefficients (*s*) towards the tested compound–target pair. Let x = (c,t) be the new test sample for which we choose a subset of *X* by retrieving $C\subset C^{t}$, |C| = k, such that $s(c^{\left(i\right)},c)\geq s(c^{\left(j\right)},c)$ holds $\forall c^{\left(i\right)}\in C$ and $\forall c^{\left(j\right)}\in C^{t}\backslash C$. Equivalently, we define $T\subset T^{t}$, a subset of targets such that |T| = q. *k* and *q* are tuneable hyperparameters, determining the neighbourhood of the tested x = (c,t) pair in the training set *Z*. Dynamic calibration set for the new test instance *x* is then defined as: where each $x^{\left(ij\right)}$ is actually a tuple $\left(c^{\left(i\right)},t^{\left(j\right)}\right)$. The dynamical calibration set defined in this manner represents the most conforming part of the training set bioactivity space with respect to the tested compound–target pair. To allow forming dynamic calibration sets from training samples, we train the model over the entire dataset by applying 10 × 10-fold cross-validation and calculate non-conformity scores for each training sample as a difference of the mean of cross-validation predictions and the true labels (Vovk *et al.* 2018). We call the proposed approach dynamic applicability domain (dAD). In the following section, we define the two alternative non-conformity scores.


(2) $$Z^{c}=\{(x^{\left(ij\right)},y^{\left(ij\right)}):x^{\left(ij\right)}\subset (C,T)\;\mathrm{and}\;\exists y^{\left(ij\right)}\subset Y^{t}\},$$

### 2.1 Definition of calibration and test non-conformity scores

In this work, we define and test two alternative formulations of non-conformity scores for the compound–target pairs from the dynamic calibration set. The first variant, dAD (NN), non-conformity score $\alpha_{i}^{nn}$ (3) is calculated by taking the difference between the experimental binding affinity of each pair in the $Z^{c}$ and mean label value for all pairs in $Z^{c}$. In alternative formulation, dAD (CV), non-conformity score $\alpha_{i}^{cv}$ (4) is based on a difference between the experimental binding affinity from the mean of 10 × 10-fold cross-validation predictions for each pair. For the test sample, the putative non-conformity scores are defined as the difference of the predicted label ($\hat{y}$) and each experimental compound–target pair binding affinity in the dynamic calibration set, $\alpha^{x}$ (5). Thus, for every new test instance *x* we get the corresponding calibration non-conformity vectors, $S^{cv}$ or $S^{nn}$ and its own vector of non-conformity scores, $S^{x}$.

Definitions of non-conformity scores associated with the test sample and its dynamic calibration set are given below: where $y_{i}^{\mathrm{cal}}$ is a true value for *i*’th sample in calibration set; ${\hat{y}}_{i}^{nn}$ and ${\hat{y}}_{i}^{cv}$ are predictions for the *i*’th sample in dAD (NN) and dAD (CV) approaches, respectively; ${\hat{y}}^{x}$ is a prediction for test sample *x*.


(3) $$\alpha_{i}^{\mathrm{cal}}=\alpha_{i}^{nn}=|y_{i}^{\mathrm{cal}}-{\hat{y}}_{i}^{nn}|,\alpha_{i}^{nn}\in S^{nn},$$

(4) $$\alpha_{i}^{\mathrm{cal}}=\alpha_{i}^{cv}=|y_{i}^{\mathrm{cal}}-{\hat{y}}_{i}^{cv}|,\alpha_{i}^{cv}\in S^{cv},$$

(5) $$\alpha_{i}^{x}=|y_{i}^{\mathrm{cal}}-{\hat{y}}^{x}|,\alpha_{i}^{x}\in S^{x},$$

In the next step, we have to find the true prediction region for a predefined confidence level for the test sample *x*. For that purpose, minimal value from the calibration non-conformity scores $\alpha_{i}^{\mathrm{cal}}$ is found, for which the expression below holds as in Shafer and Vovk (2008) and Johansson *et al.* (2014): where $z_{i}\in Z^{c}$ are samples from the calibration set. We annotate minimal value of $\alpha_{i}^{\mathrm{cal}}$ for which above expression (6) holds, $\alpha_{\delta }^{\min}$. Then, the prediction region for any new example is defined as $\Gamma_{x}^{\delta }$ = $\hat{y}\pm \alpha_{\delta }^{\min}$. As one can notice, for any predefined level of confidence, dAD produced prediction region varies between test samples as the calibration sets are sample specific.


(6) $$\frac{\#\{z_{i}\in Z^{c}|\alpha^{x}\leq \alpha_{i}^{\mathrm{cal}}\}}{N_{Z^{c}}}\geq 1-\delta ,$$

Chosen $\alpha_{\delta }^{\min}$ represents the partition of samples in the $S=S^{cv}$ or $S=S^{nn}$ that have higher non-conformity scores than any given $\alpha_{i}^{x}\in S^{x}$. Since tentative labels for each tested pair *x* are based on dynamic calibration set samples, and reflect the local model performance, no normalization step is necessary and individual prediction regions are directly inferred from $\alpha_{\delta }^{\min}$. The pseudocode of the algorithm for the construction of dynamic calibration set and calculation of non-conformity scores is given in Supplementary Algorithm S1.

### 2.2 Normalized non-conformity measures

Approaches like Papadopoulos (2008), Johansson *et al.* (2014) and Alvarsson *et al.* (2021), rely on fixed calibration sets and for that reason prediction regions these methods output are not sample specific and do not reflect the local non-conformity of test samples. In order to achieve more informative prediction regions for an individual test sample Papadopoulos *et al.* (2011) and Papadopoulos and Haralambous (2010) introduced different normalization measures.

In Shafer and Vovk (2008), non-conformity score (α) is calculated as the absolute difference of the predicted and the true value, Equation (7) in Table 1. Normalization approach as in Equation (8) requires an extra model to estimate the accuracy of individual predictions, $\mu_{i}$. On the other hand, in Equation (9), non-conformity scores are normalized by dividing it with a factor representing normalized distances of nearest neighbours ($\lambda_{i}^{k}$)—or by $\xi_{i}^{k}$, factor representing normalized standard deviation of nearest neighbours of sample *x*, Equation (10).

**Table 1.** Non-conformity measure (α) as used per four different reference studies, with included normalization coefficients.^a^

Reference	Non-conformity measure	Normalization coefficient	Eq.
Shafer and Vovk (2008)	$\alpha_{i}=|y_{i}-\hat{y_{i}}|$		(7)
Papadopoulos and Haralambous (2010)	$\alpha_{i}=\frac{\left|y_{i}-{\hat{y}}_{i}\right|}{\;\text{exp}(\mu_{i})}$	$\text{exp}(\mu_{i})$	(8)
Papadopoulos *et al.* (2011)	$\alpha_{i}=|\frac{y_{i}-{\hat{y}}_{i}}{\gamma +\lambda_{i}^{k}}|$	$\lambda_{i}^{k}$	(9)
Papadopoulos *et al.* (2011)	$\alpha_{i}=|\frac{y_{i}-{\hat{y}}_{i}}{\gamma +\xi_{i}^{k}}|$	$\xi_{i}^{k}$	(10)

a $\text{exp}(\mu_{i})$ represents model accuracy estimate ensuring always a positive value; $\lambda_{i}^{k}$ coefficient is based on distances; and $\xi_{i}^{k}$ is based on standard deviation of sample $x_{i}$ and its *k* nearest neighbours. γ is a sensitivity parameter in control of the sensitivity to changes in both $\lambda_{i}^{k}$ and $\xi_{i}^{k}$ measures.

## 3 Data

Validity of the proposed approach, is tested over several publicly available databases as shown in Table 2. Furthermore, we combine all mentioned datasets into a single dataset covering larger space of kinase inhibitors over human kinome space. Aside from the kinase inhibitors, we also retrieve the G protein-coupled receptors (GPCR) and selective serotonin re-uptake inhibitor (SSRI) subsets from the Drug Target Commons (Tang *et al.* 2018) database, in order to test the performance across more diverse bioactivity spaces. Data preprocessing involved several preprocessing filters ensuring the final datasets contained no duplicates and only those bioactivity profiles measured over the human kinome superfamily narrowing the potential protein target space down to nine distinct kinase groups, as given in Fig. 2.

**Table 2.** Publicly available databases used for compound–target binding affinity prediction, including Davis (Davis *et al.* 2011), KIBA (Tang *et al.* 2014), BindingDB (Gilson *et al.* 2016), ChEMBL (Gaulton *et al.* 2012), and DTC (Tang *et al.* 2018) and a SCKBA dataset, we constructed from the mentioned databases for the purposes of a unified representation of this specific bioactivity space.

Dataset	#cmpds	#trgts	#int
Davis	68	379	27 621
KIBA	2068	229	118 036
BindingDB	10 968	311	25 674
ChEMBL	11 637	235	51 360
DTC (GPCR)	1681	119	17 245
DTC (SSRI)	3640	49	19 046
SCKBA	7860	210	43 433

**Figure 1.**
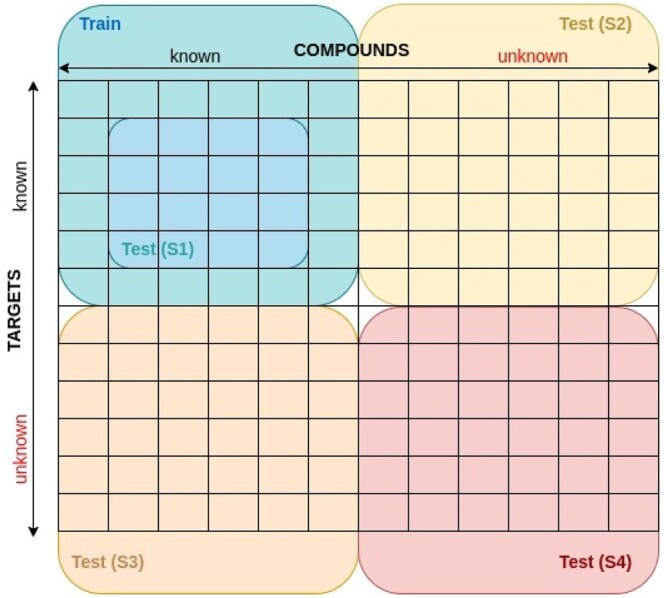
Illustration of test set construction with four difficulty levels, where S1 contains new compound–target pairs and reflects a standard way of testing and evaluating model performance by using stratified sampling and making predictions over known compounds and targets, as it was applied for all datasets in Table 2; S2 contains new compound–target pairs with compounds not available in the training set; S3 contains new compound–target pairs with targets not available in the training set; and S4 contains never seen compounds nor targets. S2–S4 scenarios were only applied for SCKBA dataset.

Acquiring larger kinase inhibitor dataset by combining available human kinome centric databases makes it possible to construct several testing scenarios. The final dataset is made sure to contain only small molecules with a molecular weight of ∼900 Da and protein targets that are members of the human kinome in order to build a more consistent small compound–kinase binding affinity dataset (SCKBA) with a well-rounded representation of the bioactivity space (Table 2). To increase the number of measured bioactivities both $K_{d}$ and $K_{i}$ were used interchangeably, as it was shown in Cichońska *et al.* (2021) that combination of bioactivity types can increase model’s overall performance. Finally, to reflect the real machine-learning use-cases with increasing levels of difficulty, the data were distributed between the training set and four different test set scenarios, as it was introduced in Cichonska *et al.* (2017) and Pahikkala *et al.* (2015), with an illustrative example in Fig. 1.

We did chemical space analysis on 7860 compounds using *t*-SNE for the generation of testing sets in this manner (Supplementary Fig. S1). Training compounds, together with their bioactivity profiles, were collected to assure high coverage of diverse compound scaffolds available in the overall compound set. The quality of the resulting compound clustering was determined by inspecting the maximum common substructures of dense clusters with a threshold of 0.7, which means that the substructure must be present in at least 70% of compounds in the chosen region. We also chose two arbitrary ‘soft clusters’, in the compound space’s middle cloud to provide us a frame of reference when deciding on specificity of common substructures. As shown in Fig. 1, first test scenario (S1) includes samples already seen in the training set, while for the second test scenario (S2), we retrieve compounds from high density regions, data points colour-coded in orange (Supplementary Fig. S1), mostly including compounds with fewer bioactivity profiles, but with many similar compounds retained in the training set. Third test scenario (S3) includes bioactivity profiles chosen based on the kinome space with fewer experimentally measured bioactivities in the overall dataset, consequently selecting compounds mostly contained within the middle cloud of compound samples, colour-coded in green (Supplementary Fig. S1). To avoid the severe reduction of the training set, for the S3 we selected only three well-known protein targets with over 200 measured binding affinities each (Supplementary Fig. S2). These protein targets include phosphoinositide-dependent kinase 1 (PDK1), checkpoint kinase 2 (CHK2), and tropomyosin receptor kinase A (TRKA), belonging to the AGC, $Ca^{2+}/$calmodulin-dependent protein kinase (CAMK), and tyrosine kinase (TK) groups, respectively. In the fourth scenario (S4), test set contains both compounds and targets that are not present in the training set, ultimately comprising of compounds from a cluster well separated from the rest of chemical space, colour-coded in red (Supplementary Fig. S1). For similar reasons as in S3, in S4 only one protein target is retained as shown in Supplementary Fig. S2, NF-κβ-inducing kinase from the STE group, with binding affinities measured over 600 different compounds. Additionally, the trained model is tested on a full test set (S0) that comprises of all samples from S1 to S4 test scenarios.

### 3.1 Compound space representation

Based on the obtained SMILES structures of the chemical space Tanimoto similarity scores are calculated by performing pairwise comparison of the entire compound space based on the 2048-bit Morgan fingerprints with selected radii of 2.

For the training of GCN on the chemical space, molecules are represented as adjacency matrices between atoms, and each atom is represented as a vector of properties. Instead of using one-hot-encoded vector representation (Nguyen *et al.* 2021), we use *rdkit* library (Landrum *et al.* 2020) in Python to compute atomic attributes that include the atomic number, charge, hybridization state, number of radical electrons, number of hydrogen atoms bound, chirality, and ring membership. Deep learning approach is implemented using PyTorch (Paszke *et al.* 2019) and PyTorch Geometric (Fey and Lenssen 2019).

### 3.2 Target space representation

**Figure 2.**
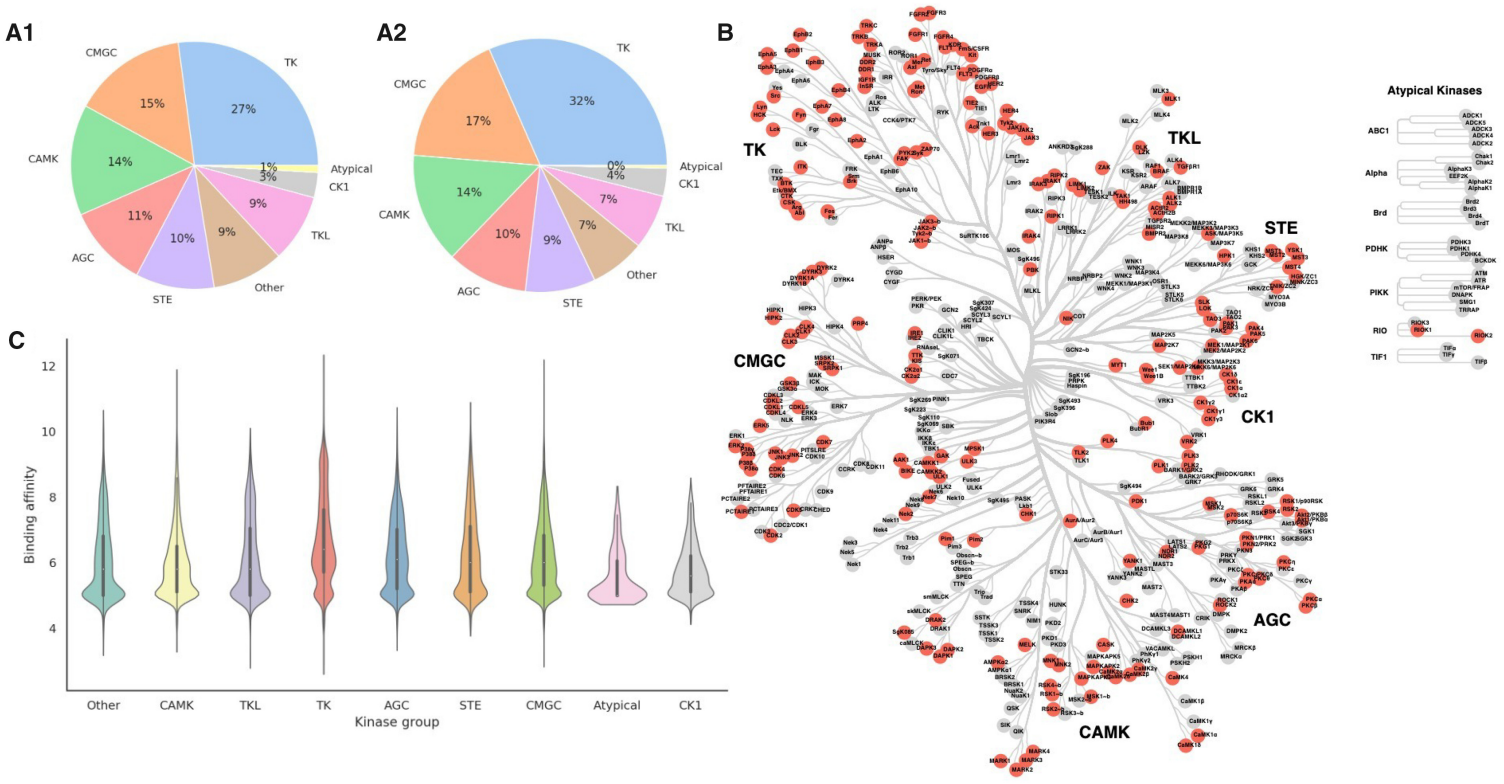
Human kinome targets available in SCKBA dataset. (A1) shows the percentage of protein targets from every kinome group available in the dataset; (A2) similarly to (A1), gives a number of compounds with measured binging affinities across different kinase groups. Complementary to the first two, (B) shows all the selected kinases and affiliated groups (Manning *et al.* 2002, Metz *et al.* 2018), and (C) shows how the experimental binding affinities (pKd and pKi) are distributed per kinase group.

The human kinome consists of ∼530 enzymes clustered into 10 smaller groups or super-families that share a common evolutionary origin (Manning *et al.* 2002, Roskoski 2015), which is best shown in Fig. 2B with selected kinases given in red. Most importantly, they catalyse phosphorylation and are included in the most important regulatory mechanisms in all living organisms (Liao 2007, Roskoski 2015). To get a better feeling for the protein targets available in the SCKBA dataset, we can check their placement in the human kinome phylogenetic tree (Fig. 2B), same as the number of kinases in any of nine dedicated kinase groups in the collected dataset (Fig. 2A1) or number of measured binding affinities per kinase group (Fig. 2A2), which tells us just how prevalent certain kinase groups are as targets, e.g. TK group, CMGC group including mostly proline-directed serine/threonine kinases, and CAMK group comprise over 50% of all protein targets in our dataset. Moreover, Fig. 2C depicts the distribution of experimentally measured binding affinities across kinase groups.

Local similarities between these protein targets are computed by applying the Smith–Waterman algorithm to the protein kinase (PK) sequences with default parameters of the *protr* library in *R* (Xiao *et al.* 2015) (gap.opening = 10, gap.extension = 4) and ‘BLOSUM62’ substitution matrix. Same approach was performed for computation of sequence similarities of the GPCR and SSRI datasets, retrieved from the DrugTargetCommons database (Tang *et al.* 2018). Specifically for the SCKBA dataset, we compute local similarities only for the PK domain, because it is highly conserved and is a major focus for the small molecule inhibitor design, with most of the approved drugs targeting exactly ATP-binding cleft or the surrounding regions (Manning *et al.* 2002, Liao 2007, Roskoski 2015). For the training of deep learning architecture (GCN–CNN) on SCKBA dataset, the same strategy is adopted as in Nguyen *et al.* (2021), treating protein targets as sequences of characters within a CNN block, with the difference of learning sequence representations by feeding the PK domains into the network, instead of using the whole protein sequences, for reasons explained above. We identified the longest PK domain sequence in our dataset and applied zero padding to other targets to match the length of the identified sequence.

## 4 Materials and methods

XGBoost is trained on all datasets from Table 2, where binding affinities are expressed as the negative logarithm of equilibrium of dissociation ($K_{d}$) or inhibition ($K_{i}$) constants, whereas GCN–CNN architecture is applied only for the SCKBA dataset.

### 4.1 XGBoost

It is a boosting ensemble method used mostly with decision tree algorithm that has proven to be fast and highly effective in achieving ‘state-of-the-art’ results on many problems (Chen and Guestrin 2016). There are several hyperparameters determining the quality and performance of the XGBoost model for a given task; we utilized grid-search approach to find the set of hyperparameters providing best performing model. Since hyperparameter tuning in this manner can become expensive, smaller subset of the SCKBA dataset was used for this purpose.

### 4.2 GCN–CNN

For the GCN–CNN approach, we started from GraphDTA architecture proposed in Nguyen *et al.* (2021). We updated it by changing node representations of compound graphs from one-hot-encoded vectors to the physicochemical atomic feature representations. Furthermore, NN architecture is customized by implementing early stopping for no significant improvement over 20 consecutive epochs, with the decrease in learning rate for every 10 epochs, in order to avoid over-fitting the model on the training data (Supplementary Fig. S7). GCN–CNN approach is trained for the total of 260 epoch with starting learning rate of 0.0005.

### 4.3 CP

Computation of calibration scores for any of the baseline approaches, Table 1, we use *Python* library ‘nonconformist’. We tune a gamma sensitivity parameter for each dataset individually in the standard CP framework. Using the narrowest median prediction region ($\alpha_{\delta }$) as a reference, we picked an appropriate value for γ under the restriction that the mean error rate does not exceed the mean of the maximum error rates while still retaining the validity of the prediction regions for each confidence level (Supplementary Figs S9 and S10).

**Figure 3.**
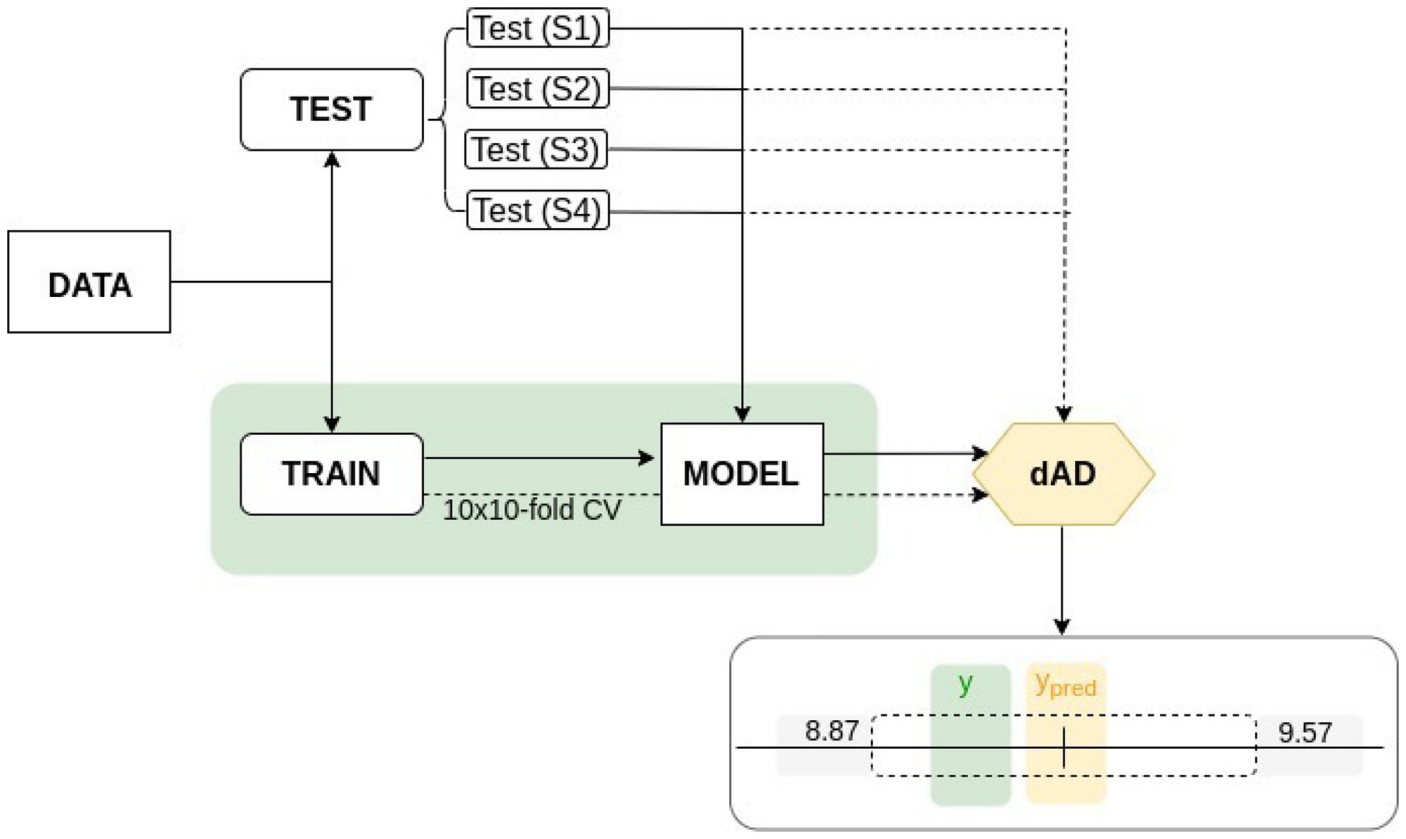
Illustration of the proposed dAD workflow from training of the underlying model to CP and validity evaluation. Data are split on train and test set(s), with all training samples being used for the modelling and allocation of the conformity region for each individual sample in the test set resulting in individual prediction regions. For a given confidence level error rate is defined by the number of samples for which the true labels (*y*) are not in the CP defined prediction region of $y_{\mathrm{pred}}$ (single CP prediction region is illustrated above as [8.87, 9.57]). Conformal predictor is valid if its error rate does not exceed 1-confidence.

As it is shown in Fig. 3, in order to assess the validity of the confidence levels (75%, 80%, 85%, 90%, 95%, and 99%), the trained model is subjected to four different levels of testing difficulty. In order to compare proposed approach with baseline studies, we implement normalized non-conformity scoring as proposed in Papadopoulos and Haralambous (2010) and Papadopoulos *et al.* (2011). For normalizing the non-conformity scores by additional error model, instead of training NN (Papadopoulos and Haralambous 2010) or random forest (Johansson *et al.* 2014), in this work, we train an XGBoost model with the same hyperparameters as the model trained to predict binding affinities. dAD uses model trained on all samples and defines a dynamic calibration set such that for each test sample compound–target pair *x*, *k*, and *q* of the most similar compounds and protein targets, respectively, are selected from the training space, with respect to the tested compound–target pair. Both hyperparameters, *k* and *q*, were tuned manually for the SCKBA test (S1) dataset, with the aim of inspecting the impact on the validity and size of the prediction regions (Supplementary Fig. S8). For the majority of cases with larger number of different compounds and targets, we used *k * = * *250 for nearest neighbours in the compound space and *q * = * *25 for nearest neighbours in the target space, with the exception of Davis (Davis *et al.* 2011) and SSRI (Tang *et al.* 2018) datasets. Davis consists of 68 compounds in total and SSRI consists of only 49 protein targets, so smaller values for *k* and *q* were used, *k * = * *25 and *q * = * *10, respectively.

### 4.4 CP evaluation

Conformal Predictor framework is a model agnostic approach and addresses the uncertainty of the predictions. It calibrates model predictions in terms of some predefined confidence level using the calibration set of samples, and provides uncertainty assessment on an individual prediction level: for each model prediction, it provides prediction region where the true value should lie (Shafer and Vovk 2008). The CP diagnostics typically addresses validity and efficiency (informativeness) of the conformal predictor over different levels of confidence. This is also the basis of our assessment of dAD framework and comparisons against other CP frameworks. Validity of conformal predictors is assessed through error rate defined as the ratio of tested samples whose predictions do not fall into prediction region for certain confidence level, where expected error is ∼1-confidence. Conformal predictor is said to be valid if observed error rate is smaller or equal the expected error. Informativeness is another measure used to assess conformal predictors. It is related to the size of the prediction region for certain level of confidence. For the regression problem and CP assessment the size of the prediction region for the particular sample is defined by the value of non-conformity score (α) (see Section 2.1). To test how well the conformal regressor can recognize true binding affinities and produce valid prediction regions, the share of falsely classified compound–target pairs is determined for each confidence level of the four test scenarios in SCKBA dataset (Table 4). Due to the variable size of dynamic calibration sets and the difference in distribution of calibration and test sample non-conformity scores, dAD does not guarantee the extraction of $\alpha_{\delta }^{\min}$ scores for every sample at any confidence level. This ‘abstaining from prediction’ property is a direct result of introducing putative test non-conformity scores, as described in Section 2.1. For that reason, we denote the level of coverage (% of samples with prediction) for every confidence level as a value in addition to the reported error rates. Also, for a more direct comparison between the methods, we report the results only for those compound–target pairs and confidence levels that dAD (NN) and dAD (CV) methods were able to produce (Supplementary Tables S1, S2 and S4). Ultimately, dAD is compared to the previous studies by inspecting the median α range and mean error rates over all confidences levels. How well the calibration set represents the test sample is completely up to the representation of samples in the training set regarding the distance of nearest neighbours and number of experimentally measured binding affinities. Accordingly, following the work of Kuleshov *et al.* (2018) and Levi *et al.* (2022), we compare how well calibrated our proposed approach is for different testing scenarios and when compared to the baseline (Supplementary Figs S12 and S13).

**Table 4.** Comparison of baseline methods with proposed dAD approach on a combined SCKBA dataset over four difficulty scenarios, with sensitivity parameter γ for Equations (9) and (10) in S1 scenario being $\gamma_{\left(\lambda \right)}$=0.2 and $\gamma_{\left(\xi \right)}$=0, respectively, and 0 for the rest.^a^

SCKBA									
		Median		Error rates per confidence level (%)					
Approach	SX	$\alpha_{\delta }$	#calib	75%	80%	85%	90%	95%	99%
Shafer & Vovk (7)	S0	0.86	4000	44.69	40.39	35.90	29.81	22.87	12.26
	S1	0.86	4000	21.83	16.34	11.91	6.56	3.66	0.76
	S2	0.86	4000	40.64	35.08	30.27	21.60	12.41	2.57
	S3	0.86	4000	35.49	31.73	26.02	18.95	11.43	3.91
	S4	0.86	4000	86.17	84.50	81.83	80.00	72.83	49.17

Papadopoulos (8)	S0	1.94	4000	27.18	23.64	20.49	16.04	9.88	1.61
	S1	2.45	4000	5.65	4.12	3.05	2.14	1.53	0.31
	S2	1.65	4000	22.35	18.93	15.72	10.48	5.99	1.39
	S3	2.38	4000	11.13	7.82	5.11	3.61	2.56	0.30
	S4	1.62	4000	76.00	69.83	64.00	53.67	33.17	4.83

Papadopoulos (9)	S0	1.64	4000	25.43	19.58	15.13	9.56	5.15	1.02
	S1	1.02	4000	19.69	15.73	10.84	7.33	5.50	1.68
	S2	1.13	4000	30.16	24.39	19.68	13.69	7.49	1.39
	S3	1.55	4000	22.71	18.35	14.74	10.68	5.86	1.20
	S4	3.23	4000	33.83	25.17	17.17	6.33	2.67	0.00

Papadopoulos (10)	S0	0.85	4000	45.25	40.56	36.50	31.17	25.08	14.43
	S1	0.85	4000	22.60	16.49	12.98	7.79	4.12	1.22
	S2	0.85	4000	43.10	37.75	32.09	25.67	17.11	5.67
	S3	0.95	4000	32.18	27.37	22.71	16.54	10.53	3.01
	S4	0.78	4000	87.83	85.83	84.33	81.50	76.5	55.17

dAD (NN)	S0	1.77	259	13.14 (0.63)	17.97 (0.64)	14.00 (0.67)	15.36 (0.72)	12.38 (0.75)	3.69 (0.69)
	S1	1.85	315	1.87 (0.73)	1.65 (0.74)	0.79 (0.77)	1.00 (0.76)	0.62 (0.74)	0.00 (0.63)
	S2	1.78	253	8.76 (0.74)	6.72 (0.70)	3.54 (0.63)	2.94 (0.58)	1.28 (0.59)	0.36 (0.60)
	S3	1.65	279	11.90 (0.69)	10.11 (0.71)	8.07 (0.73)	6.03 (0.77)	4.76 (0.79)	1.03 (0.73)
	S4	1.79	232	70.70 (0.26)	68.40 (0.38)	60.47 (56)	52.27 (0.84)	39.22 (0.98)	12.69 (0.87)

dAD (CV)	S0	1.57	259	14.90 (0.54)	16.62 (0.55)	16.85 (0.54)	18.98 (0.55)	14.59 (0.46)	2.81 (0.28)
	S1	1.57	315	2.04 (0.60)	2.13 (0.57)	1.13 (0.54)	1.37 (0.45)	0.47 (0.33)	0.00 (0.19)
	S2	1.55	253	10.37 (0.58)	7.47 (0.56)	4.87 (0.48)	3.54 (0.42)	1.41 (0.42)	0.77 (0.28)
	S3	1.46	279	12.97 (0.60)	11.68 (0.60)	9.24 (0.55)	7.36 (0.55)	5.90 (0.41)	0.00 (0.18)
	S4	1.74	232	71.23 (0.24)	68.60 (0.34)	59.67 (0.51)	53.47 (0.72)	42.96 (0.70)	8.15 (0.45)

a The SX denotes the testing scenario; $\alpha_{\left(\delta \right)}$ is the median prediction region of the test set; #calib is number of samples in the calibration set or median number of samples for the dAD method with varying calibration sizes. Error rates represent the percent of samples with labels outside of prediction regions. Values next to the dAD (CV) and dAD (NN) error rates denote the coverage of the test set, with 0 meaning that the proposed approach was not able to produce prediction regions for a given confidence and 1 meaning that it produced a prediction region for every test sample.

## 5 Results

### 5.1 Choosing the prediction algorithm and model

We tested two different algorithms and problem representations to produce prediction models for testing and comparison of CP approaches over all datasets. As a conventional and faster approach, XGBoost was used as a baseline method. In addition, GCN–CNN architecture is adapted and applied, as a ‘state-of-the-art’ deep representation learning approach. We start with the architecture from Nguyen *et al.* (2021) as explained in Section 4. In Table 3, we can see that XGBoost approach is comparable, if not better in performance than the more complex convolutional network. This outcome may be unique to this dataset, where extended circular fingerprints capture chemical structure variation in the compound space sufficiently well for the model to generalize to real-world scenarios (S2 and S3). Furthermore, it is possible that the number of samples required for a robust NN model was not large enough, so by training over larger datasets with pretrained embeddings, GCN–CNN could be boosted in terms of scoring metrics, but this was beyond the scope of this study.

**Table 3.** XGBoost and GCN–CNN trained on a SCKBA train set with 40 578 interactions between 6325 compounds and 206 PKs.^a^

Test				XGBoost		GCN–CNN	
SX	#int	#cmpds	#trgts	MSE	CI	MSE	CI
S0	2855	2170	181	1.24	0.74	1.49	0.70
S1	655	411	169	0.28	0.83	0.43	0.82
S2	935	935	59	0.61	0.79	0.95	0.75
S3	655	439	3	0.60	0.72	0.61	0.74
S4	600	600	1	3.97	0.53	3.74	0.53

a Models are tested and compared over (S0) test set, including four difficulty scenarios (S1–S4) obtained by segmentation of the S0 dataset.

### 5.2 Comparison over different difficulty scenarios

As shown in Table 4, the dAD approach exhibits lower error rates per confidence level, in comparison to the standard approaches. The performance gap is most pronounced in the testing scenarios S2 and S3, which include compounds and targets that were not seen during the training phase, respectively. Poorer performance in these two settings could be due to the fact that using one-fits-all calibration approach confines the hypothetical prediction value between strict upper and lower boundaries that do not generalize well when it comes to unseen samples. Significant difference can also be observed on S0 test scenario, where both Equations (8) and (9) produce valid prediction regions for every confidence level, but with slightly larger prediction regions on average than dAD variants.

**Figure 4.**
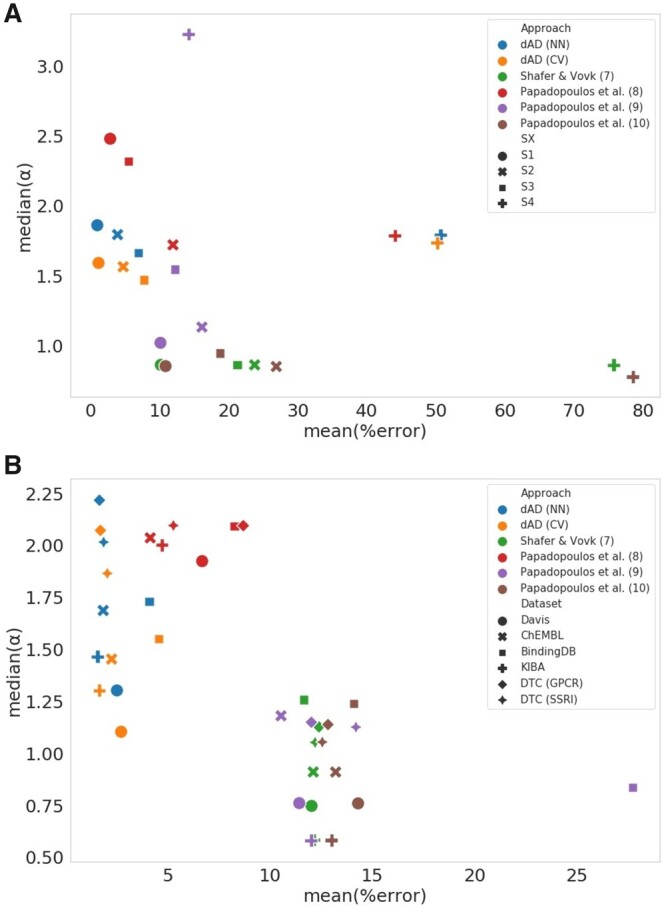
Comparison of the proposed dAD approach with the baseline studies by showing the relationship of mean (%) error and median non-conformity scores for (A) four different testing scenarios (S1–S4) of SCKBA dataset and (B) over six compound–target datasets, Davis (Davis *et al.* 2011), KIBA (Tang *et al.* 2018), ChEMBL database (Gaulton *et al.* 2012), BindingDB (Gilson *et al.* 2016), DTC (GPCR) (Tang *et al.* 2018), and DTC (SSRI) (Tang *et al.* 2018) represented with different shapes.

In the testing scenario S1, all approaches seem to be equally effective in keeping the number of incorrectly classified samples within proper limits. In contrast, in the testing scenario S4, neither approach is effective, and all of them show a high number of incorrectly classified samples. Considering that protein targets in S3 and S4 test sets belong to the kinase groups with many representatives in the training set, we assume that the skewed performance is due the variability in the compound space, especially in the S4 setting where the *t*-SNE analysis shows that all compounds belong to the cluster separate from the rest of the training samples. Supplementary Fig. S3 depicts how median of the prediction region (α) varies for different CP approaches; Shafer and Vovk (7) method is represented simply as a point, due to the fact that prediction region is constant for all test samples at given confidence level. Median prediction regions for dAD (NN) and dAD (CV) are comparable, and range from one to three units depending on the confidence level. Variation across methods, in terms of prediction regions remains similar even when only dAD covered test samples are taken into account (Supplementary Fig. S4). However, we cannot make the assumption that one method is superior to another simply by looking at the error rates or the validity of a conformal predictor; but rather also consider the width of the prediction regions for each of these test cases. A good conformal predictor should strike a balance between both of these measures, staying valid while ensuring that prediction regions are as narrow as possible. Large prediction regions, while ensuring validity for any test scenario, are not useful. It is well illustrated in Fig. 4A (Supplementary Fig. S11A), where all six CP approaches are compared by examining the relationship of the mean error rates with the median $\alpha_{\delta }$ scores of paired and unpaired datasets (Supplementary Table S1).

Results of both dAD (NN) and dAD (CV) seem to be comparable. Similar case is with the Shafer and Vovk approach and Papadopoulos (10), showing somewhat tighter prediction regions but higher error rates on average than the proposed dAD approach. In Fig. 4A, we visualize performance of all six methods over four different testing scenarios (S1–S4) by plotting the relationship between the mean error rates and median prediction region width (α). For S1 test setting, denoted as points in Fig. 4A, all methods besides Papadopoulos (8) in red, give reasonably tight prediction regions with maximum median prediction region lower than two units and with low error rates, which is to be expected due to the nature of that testing scenario comprising of compound and PK targets already seen during the training phase. At the other end of the spectrum, there is an S4 test setting where all trained models show almost random performance, as we see in Fig. 4A where the performance over the S4 test set marked as pluses occupies the right end of the scatter plot. Papadopoulos (9) is an exception, achieving very low mean error rate on the forth scenario due to the very wide median prediction region. Both proposed dAD approaches show low mean error rates with relatively tight prediction regions, which is especially important for more realistic test scenarios, S2 and S3.

Calibration quality of dAD is inspected in terms of expected and observed confidence levels, and in comparison to baseline studies. We additionally investigate whether and how calibration quality changes with the addition of a normalization measure similar to Equation (9). In terms of calibration, we can say that dAD exhibits underconfident results, with the observed confidence always being higher than the expected one, which is opposite of how the baseline approaches behave. Even if in real use-case scenarios, an underconfident estimator would be preferred over an overconfident one—we inspect if the both observed and expected confidence could be brought to a closer agreement. Introducing a normalization measure leads to a reduction in the width of selected prediction regions (Supplementary Table S5). As a result, the observed confidence decreases, causing the dAD to become overconfident for higher confidence levels in S2 and S3. Moreover, this normalization makes the approach even less reliable in cases where it was initially overconfident, such as S4. It is best shown in Supplementary Fig. S12, where the effect of normalization evidently draws observed and expected confidence level closer to a certain point. Furthermore, Supplementary Fig. S13 reveals that baseline approaches exhibit good calibration on average for the S1 testing scenario. However, in the other testing scenarios, they tend to yield higher expected confidence than the observed confidence, leading to overconfident predictions.

### 5.3 Comparison over standard benchmark datasets

Table 5 and Supplementary Tables S3 and S4 compare the four conformal predictor approaches with two proposed dAD variants. Shafer and Vovk approach (Shafer and Vovk 2008) shows lower validity of predicted regions when compared to the normalization based CPs or the proposed approaches. This behaviour is expected, especially since it produces fixed prediction regions (α scores) given the confidence level. Having this in mind, even if the mean of the prediction region is lower than in any other approach, true difference is shown in the validity of their predictions.

**Table 5.** Comparison of baseline methods with proposed dAD approach on compound–kinase binding affinity datasets from Table 2, with sensitivity parameter γ of Equations (9) and (10) for the Davis and KIBA datasets being $\gamma_{\left(\lambda \right)}$ = 0.7 and $\gamma_{\left(\xi \right)}$ = 0; for BindingDB dataset $\gamma_{\left(\lambda \right)}$ = 0 and $\gamma_{\left(\xi \right)}$ = 0; and for the ChEMBL dataset $\gamma_{\left(\lambda \right)}$ = 0.3 and $\gamma_{\left(\xi \right)}$ = 0.^a^

Benchmark datasets (KI)									
		Median		Error rates per confidence level (%)					
Dataset	Approach	$\alpha_{\delta }$	#calib	75%	80%	85%	90%	95%	99%
Davis	Shafer & Vovk (7)	0.75	1500	23.86	19.28	14.43	9.77	4.13	0.76
	Papadopoulos (8)	1.92	1500	13.12	10.36	7.63	5.32	2.76	0.91
	Papadopoulos (9)	0.76	1500	23.13	18.46	13.61	9.03	3.73	0.65
	Papadopoulos (10)	0.76	1500	26.08	21.77	17.61	12.78	6.28	1.36
	dAD (CV)	1.10	502	3.56 (0.34)	3.33 (0.52)	3.30 (0.71)	3.04 (0.80)	2.21 (0.77)	0.87 (0.46)
	dAD (NN)	1.30	502	3.43 (0.33)	3.25 (0.52)	3.24 (0.71)	2.81 (0.83)	1.87 (0.91)	0.48 (0.91)
KIBA	Shafer & Vovk (7)	0.58	3000	23.96	19.08	14.73	9.41	4.79	0.94
	Papadopoulos (8)	2.00	3000	8.65	6.92	5.55	3.82	2.34	1.00
	Papadopoulos (9)	0.58	3000	23.62	18.96	14.51	9.42	4.66	0.91
	Papadopoulos (10)	0.58	3000	24.85	20.01	15.73	10.23	5.77	1.46
	dAD (CV)	1.30	1661	3.77 (0.73)	2.62 (0.80)	1.99 (0.87)	1.04 (0.91)	0.39 (0.84)	0.09 (0.46)
	dAD (NN)	1.47	1661	3.60 (0.72)	2.46 (0.81)	1.85 (0.90)	0.96 (0.96)	0.39 (0.98)	0.1 (0.93)
BindingDB	Shafer & Vovk (7)	1.26	3000	23.36	28.85	13.08	8.84	5.00	0.84
	Papadopoulos (8)	2.09	3000	13.48	11.37	8.84	6.9	5.13	3.85
	Papadopoulos (9)	0.84	3000	37.41	34.79	31.12	27.28	23.01	12.88
	Papadopoulos (10)	1.24	3000	25.52	21.6	16.42	11.78	7.45	1.83
	dAD (CV)	1.55	133	9.06 (0.58)	6.72 (0.55)	5.45 (0.49)	3.54 (0.44)	1.86 (0.39)	0.80 (0.27)
	dAD (NN)	1.33	133	8.64 (0.73)	6.08 (0.71)	4.58 (0.68)	3.09 (0.67)	1.54 (0.65)	0.61 (0.48)
ChEMBL	Shafer & Vovk (7)	0.91	3000	24.62	19.27	13.92	9.40	4.51	0.99
	Papadopoulos (8)	2.04	3000	8.01	6.43	4.62	3.28	1.83	0.69
	Papadopoulos (9)	1.18	3000	19.79	16.01	12.13	8.77	4.91	1.66
	Papadopoulos (10)	0.91	3000	25.41	20.49	15.13	10.63	5.86	1.77
	dAD (CV)	1.45	253	5.18 (0.74)	3.70 (0.68)	2.35 (0.62)	1.47 (0.53)	0.62 (0.40)	0.00 (0.15)
	dAD (NN)	1.69	253	4.38 (0.86)	3.11 (0.86)	1.85 (0.86)	1.13 (0.86)	0.43 (0.83)	0.15 (0.57)

a Column definitions are the same as in Table 4.

On the other hand, normalization using the underlying error model and $\lambda_{i}^{k}$ coefficient gives comparable results to the proposed dAD method for 95% and 99% confidence levels on the Davis dataset. The difference is better depicted in boxplots in Supplementary Figs S5 and S6 showing the distribution of prediction regions for every *x* in the test set, and only over samples for which dAD methods were capable to define prediction regions, respectively.

True difference in the performance of any of mentioned methods is shown by scatter plot giving relation of the mean percentage of wrongly classified samples to the median α score for confidence levels 75%–99% (Fig. 4B and Supplementary Fig. S11B), where the better performing CPs should be closer to zero on both axes. When compared in this sense, approaches (7), (9), and (10) have the narrowest prediction regions when tested on all six datasets, consequently with higher mean error rates ranging above 10%. When taking into consideration both the prediction regions and mean error rates, dAD (NN) and dAD (CV) produce more optimal prediction regions in relation to the mean error rate (Fig. 4B), with median prediction regions exceeding the two units only for the DTC (GPCR) and DTC (SSRI) datasets.

### 5.4 Demonstration of the direct application of dAD

**Figure 5.**
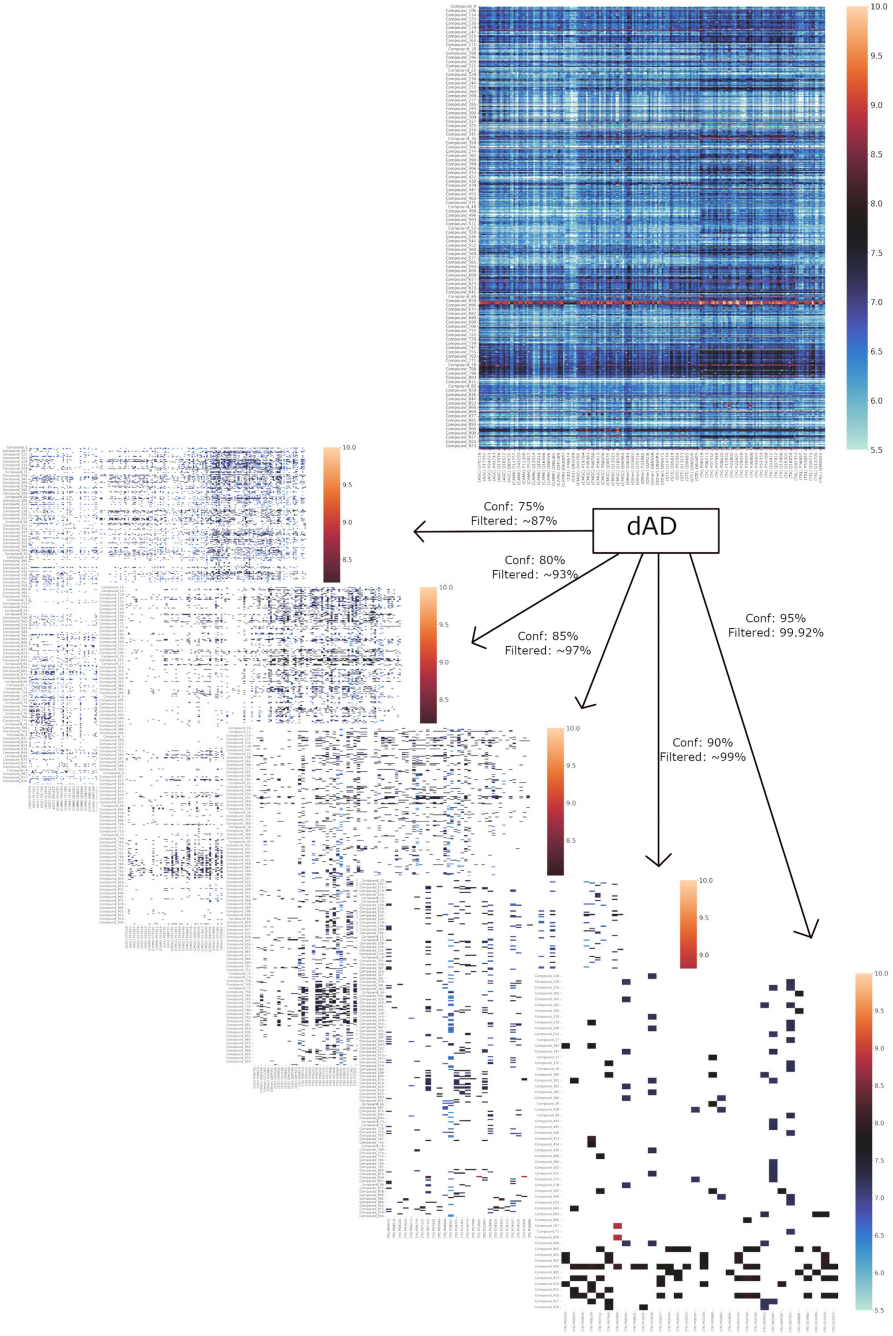
dAD as a screening filter for model predictions: first heatmap represents a subset of model predictions for all permutations of S2 compounds and protein targets in the training set, with pKd predictions ranging from 5.5 to 10 units. After applying the dAD, interaction landscape is condensed based on the chosen confidence level—there are five versions of the same heatmap depending on the dAD confidence filter applied; for confidence of 75%—13% original samples remains; 80%—7% of original samples remains; confidence of 85%—3% of original test samples remains in the heatmap, etc.

The dAD, as defined in this study, provides range-to-point predictions with a certain level of confidence. However, how does it help us condense the wide field of possible interactions? We demonstrate this on the S2 test set, one of the more difficult scenarios, by permuting all S2 test set compounds across the protein targets in the training set. This yields the heatmap depicted in Fig. 5. In the upper portion of Fig. 5, a heatmap depicts all predicted binding affinities with pKd ≥ 5.5, as generated by the trained model, while five heatmaps represent the outcomes of dAD confidence filters. Considering that binding affinities with measured pKd around six units are considered significant for the drug–kinase interaction problem and taking into account highly unbalanced data, the original filter is set up to retain only those interactions with pKd ≥ 5.5 and with prediction regions for a certain confidence level that do not exceed the lower boundary of activity. Results are displayed for confidence levels of 75%, 80%, 85%, 90%, and 95%, and for each level, only 13%, 7%, 3%, 1%, and 0.08% of samples, respectively, are filtered through as potentially significant, drastically reducing the noise in the interaction space and condensing the interaction landscape of interest. The filter criteria can be determined based on the available data and the acceptable prediction region width as deemed appropriate by the end-user.

## 6 Discussion

This work merges concepts of an AD and conformal predictors to provide improved estimates for the bioactivity prediction tasks, involving complex interaction data spaces. ‘State-of-the-art’ CP frameworks rely on fixed calibration sets based on their overall distributional similarity to the label space of the training data. This is a limiting factor, especially considering the compound–target binding affinity problem is defined by duality, independently distributed chemical and biological spaces—and models produced over such datasets may perform differently over distinct subspaces of compounds and target families depending on their distribution in the training set. Moreover, in drug discovery and repurposing, machine-learning models must predict novel compounds or targets for which exchangeability is only marginally satisfied. This was a motivation for developing novel conformal predictor framework. We solve these problems by defining calibration sets separately for each tested sample, taking into account data distribution from both ends, and then retrieving experimentally measured binding affinities for existing interaction pairs. Consequently, this method produces prediction regions that are specific for the particular test sample, given the confidence level of interest.

We prove in this work that our CP approach more accurately reflects the performance of the model in the area close to the tested sample, providing more robust prediction region estimates for any given confidence. On the standard testing scenario (S1) our experiments show that this approach provides similar performance in terms of validity and size of prediction regions as other ‘state-of-the-art’ CP approaches (Papadopoulos and Haralambous 2010, Papadopoulos *et al.* 2011). However, for more difficult scenarios (S2–S4), involving test samples at or beyond the borders of the training data space, proposed dAD approach proved to be more effective, providing strong validity with reasonable sizes of prediction regions. These findings imply that a dynamically defined CP calibration strategy more precisely reflects biases of the trained models in the neighbourhood of tested points. In terms of practical impact of these findings for biochemical research, this methodology should lead to more efficient experimental research by reducing number of false positives when screening for novel prospective drugs. As the dAD framework provides range predictions with more accurate uncertainty assessment, it is crucial for more efficient prioritization of (expensive) experiments (e.g. selection of the most promising hit molecules from *in silico* screening or drug repurposing experiments). Specifically, we demonstrate that it is more effective in realistic modelling use-cases in which predictions are made at the model’s AD boundary. We should acknowledge several caveats related to dAD approach. While providing better uncertainty calibration and prediction region estimates for realistic use-cases, due to the smaller sizes of calibration sets and novel algorithm for the determination of non-conformity scores of tested samples, dAD approach can abstain from prediction for some of the tested samples at required confidence level. This property is the consequence of our definition of non-conformity scores for test samples and reduced size of the calibration sets, and is not observed for the other CP approaches. In Supplementary Fig. S14, we demonstrate this effect and analyse the joint and individual impacts of the non-conformity definition and reduced calibration set size. Supplementary Fig. S14A illustrates joint impact of non-conformity definition and reduced calibration set size on coverage (% of test samples with CP), showing that an increased calibration set size results in increased coverage across all confidence levels. Supplementary Fig. S14B depicts the coverage of test samples using the standard definition of non-conformity (i.e. prediction regions for the test sample are based on a calibration set sample with predefined level of confidence), thus seeing the impact of reduced calibration set size on coverage, without dAD non-conformity.

The ‘abstaining from prediction’ property of dAD results in underconfident behaviour (error rate is smaller than predicted) for the samples that obtained dAD CP at certain level of confidence. This is especially characteristic for more realistic scenarios (S2 and S3) and in contrast to other approaches which are overconfident in their prediction (error rates are higher than predicted) (Supplementary Figs S12 and S13 and Supplementary Table S1). Abstaining from prediction is a common property of the conformal predictors for classification problems. We consider it to be a positive feature, which results in lower rate of false positives in more demanding prediction settings. Another caveat is related to smaller calibration sets, which limits the use of the method to problems with larger number of samples available for training the model and subsequent dynamic calibration. However, when this requirement is satisfied (as is the case for SCKBA dataset in this work), we have shown that the method exhibits stable performance for rather broad span of calibration set sizes. Future avenues related to the dAD approach may focus on explainability aspects of individual interaction predictions by exploiting localized calibration of predictions and, by extending the approach to other biological interaction type of problems.

## Supplementary Material

btad465_Supplementary_Data
