# Supplementary material for: Dynamic applicability domain (dAD): compound–target binding affinity estimates with local conformal prediction
Source: Bioinformatics. 2023 Aug 18;39(8):btad465. doi: 10.1093/bioinformatics/btad465 (PMC10457664; doi:10.1093/bioinformatics/btad465)
Supplement: btad465_Supplementary_Data [file btad465_supplementary_data.pdf]

1 Supplementary

Algorithm 1 dAD (CV/NN)

Input: x=(c_x, t_x), δ, C, T
Output: S^{cal}, α_δ^{min}, Γ_x^δ

Step 1. Selecting k closest compounds
for each $c_j \in C$ **do**
  Compute Tanimoto similarity coefficients for c_x towards all compounds in the training set, $c_i \in C$
  Rank all compounds based on their similarity scores in descending order
  Select top k compounds from the training set (C)
end for

Step 2. Selecting q closest targets
for each $t_j \in T$ **do**
  Compute Smith-Waterman similarity coefficients for t_x towards all targets in the training set, $t_j \in T$
  Rank all targets based on their similarity scores in descending order
  Select top q targets from the training set (T)
end for

Step 3. Find experimentally measured binding affinities, $y_{ij} \in Y$, between the top ranking samples
Retrieve all (c_i, t_j, y_{ij}) tuples and construct a calibration set, such that $c_j \in C^{cal}$ and $t_j \in T^{cal}$ and $y_{ij} \in Y^t$

Step 4. Determine the prediction region of sample x, Γ_x^δ
Compute nonconformity scores for x, α^{cal} and α^x, according to Eq. (**??**-**??**)
for each $\alpha_i^{cal} \in S^{cal}$ **do**
  conf = **countif** $\alpha_j^x \in S^x \leq \alpha_i^{cal}$
  if (conf $\geq$ 1-δ) **and** ($\alpha_i^{cal} < \alpha_\delta^{min}$) **then**
    $\alpha_\delta^{min} = \alpha_i^{cal}$; $\Gamma_x^\delta = \hat{y} \pm \alpha_\delta^{min}$
  else
    α_δ^{min} = *undefined*
  end if
end for

Table 1: Comparison of baseline methods with proposed dynamic applicability domain (dAD) approach on combined drug-kinase binding affinity dataset over four difficulty scenarios. The *SX* denotes the testing scenario; $\alpha_{(\delta)}$ is the median prediction region of the test set; #*calib* is number of samples in the calibration set or median number of samples for the dAD method with varying calibration sizes. Error rates represent the percent of samples with labels outside of prediction regions. Coverage values next to the dAD (CV) and dAD (NN) error rates are not reported since all results here are matched to the indices of samples where both dAD approaches were able to produce prediction regions.

SKCBA (paired)									
		Median		Error rates per confidence level (%)					
Approach	SX	α_{δ}	#calib	75%	80%	85%	90%	95%	99%
Shafer & Vovk (7)	S0	0.78	4000	36.69	34.06	33.08	34.09	32.85	19.94
	S1	0.78	4000	20.66	13.91	11.33	5.83	3.15	0.00
	S2	0.78	4000	35.68	29.55	22.65	16.99	11.83	2.72
	S3	0.78	4000	36.60	29.14	26.83	22.35	16.10	1.77
	S4	0.94	4000	86.50	84.19	80.00	80.62	76.29	48.2
Papadopoulos (8)	S0	1.90	4000	18.79	18.80	16.18	17.21	11.27	0.82
	S1	1.98	4000	5.06	4.45	2.52	1.59	1.15	0.00
	S2	1.71	4000	17.14	15.05	8.97	7.82	5.16	1.06
	S3	1.93	4000	14.45	9.91	5.90	3.92	3.26	0.00
	S4	1.99	4000	73.68	67.00	52.35	44.13	24.11	1.14
Papadopoulos (9)	S0	1.34	4000	24.14	20.15	16.48	9.43	5.20	0.63
	S1	0.89	4000	19.11	14.44	9.70	7.93	6.37	0.00
	S2	0.88	4000	30.65	25.00	19.77	12.50	6.23	1.48
	S3	1.37	4000	26.05	22.08	18.38	11.24	5.79	0.00
	S4	3.04	4000	25.76	25.87	24.45	7.95	4.76	0.00
Papadopoulos (10)	S0	0.84	4000	35.36	33.38	33.29	33.95	34.23	22.44
	S1	0.77	4000	21.20	15.53	12.29	7.93	3.85	0.00
	S2	0.88	4000	34.39	34.43	23.58	17.62	13.91	9.38
	S3	0.85	4000	33.07	25.91	24.51	17.00	12.83	0.85
	S4	0.86	4000	86.47	84.73	82.89	80.52	79.24	52.24
dAD (NN)	S0	1.54	259	16.61	15.77	16.85	19.11	17.48	5.10
	S1	1.55	315	2.36	2.18	1.14	1.38	0.96	0.00
	S2	1.52	253	10.47	7.86	4.54	3.63	1.48	0.89
	S3	1.42	279	13.39	11.40	10.03	8.22	1.17	0.00
	S4	1.68	232	69.92	68.47	61.75	54.23	45.11	13.06
dAD (CV)	S0	1.55	259	14.54	15.56	16.30	18.63	16.42	3.35
	S1	1.56	315	2.09	2.18	1.14	1.38	0.48	0.00
	S2	1.55	253	10.09	7.47	4.76	3.63	1.48	0.92
	S3	1.46	279	13.65	11.40	9.19	7.65	6.04	0.00
	S4	1.73	232	70.68	67.98	59.73	53.05	42.96	8.21

Table 2: Comparison of baseline methods with proposed dynamic applicability domain (dAD) approach on benchmark datasets involving compound-kinase binding affinities. $\alpha_{(\delta)}$ is the median prediction region of the test set; #*calib* is number of samples in the calibration set or median number of samples for the dAD method with varying calibration sizes. Error rates represent the percent of samples with labels outside of prediction regions. Coverage values next to the dAD (CV) and dAD (NN) error rates are not reported since all results here are matched to the indices of samples where both dAD approaches were able to produce prediction regions.

Benchmark datasets (KI) [paired]									
		Median		Error rates per confidence level (%)					
Dataset	Approach	α_δ	#calib	75%	80%	85%	90%	95%	99%
Davis	Shafer & Vovk (7)	0.75	1500	23.86	19.28	14.43	9.77	4.13	0.76
	Papadopoulos (8)	2.40	1500	1.39	1.23	1.23	1.64	1.35	1.11
	Papadopoulos (9)	0.84	1500	8.93	6.24	5.50	4.56	2.75	0.83
	Papadopoulos (10)	0.82	1500	14.86	10.55	9.05	7.53	4.23	1.11
	dAD (CV)	1.11	502	3.54	3.26	3.29	3.04	2.19	0.87
	dAD (NN)	1.11	502	3.43	3.26	3.27	2.9	2.21	0.94
KIBA	Shafer & Vovk (7)	0.58	3000	23.96	19.08	14.73	9.41	4.79	0.94
	Papadopoulos (8)	2.09	3000	7.04	5.20	4.28	3.15	2.24	1.15
	Papadopoulos (9)	0.56	3000	20.02	15.58	12.28	8.38	4.65	1.26
	Papadopoulos (10)	0.57	3000	21.93	16.98	13.42	9.02	5.42	1.79
	dAD (CV)	1.31	1661	3.71	2.59	1.97	1.03	0.39	0.09
	dAD (NN)	1.32	1661	3.68	2.53	1.91	1.01	0.43	0.15
BindingDB	Shafer & Vovk (7)	1.26	3000	23.36	28.85	13.08	8.84	5.00	0.84
	Papadopoulos (8)	2.05	3000	11.27	10.19	8.07	6.4	5.00	5.8
	Papadopoulos (9)	0.57	3000	38.89	36.64	34.29	29.61	23.51	21.49
	Papadopoulos (10)	1.19	3000	22.26	18.65	13.94	8.32	6.32	3.00
	dAD (CV)	1.52	133	8.85	6.61	5.35	3.49	1.85	0.75
	dAD (NN)	1.52	133	9.28	6.84	5.35	3.49	1.79	1.03
ChEMBL	Shafer & Vovk (7)	0.91	3000	24.62	19.27	13.92	9.40	4.51	0.99
	Papadopoulos (8)	1.88	3000	7.04	5.6	4.18	2.93	2.14	0.22
	Papadopoulos (9)	0.96	3000	20.48	17.08	13.48	9.98	6.44	2.46
	Papadopoulos (10)	0.83	3000	23.86	18.89	13.92	9.34	5.45	1.09
	dAD (CV)	1.45	253	5.06	3.68	2.31	1.46	0.63	0.22
	dAD (NN)	1.44	253	5.01	3.71	2.27	1.53	0.76	0.22

Table 3: Comparison of baseline methods with proposed dynamic applicability domain (dAD) approach on DTC (GPCR) and DTC (SSRI) datasets from Table 2, with sensitivity parameter γ for Eq. 9 and 10 for GPCR dataset being $\gamma_{(\lambda)}$=0.7 and $\gamma_{(\xi)}$=0; and for SSRI dataset $\gamma_{(\lambda)}$=0 and $\gamma_{(\xi)}$=0. Column definitions are the same as in Table 4.

DTC (GPCR; SSRI)									
		Median		Error rates per confidence level (%)					
Dataset	Approach	α_δ	#calib	75%	80%	85%	90%	95% CI	99%
GPCR	Shafer & Vovk (7)	1.13	1500	25.02	18.85	14.29	10.00	5.16	1.07
	Papadopoulos (8)	2.09	1500	13.62	11.24	9.02	7.46	6.02	4.82
	Papadopoulos (9)	1.15	1500	24.15	18.53	14.29	9.19	5.13	0.87
	Papadopoulos (10)	1.14	1500	25.28	18.93	14.93	10.44	6.18	1.28
	dAD (CV)	2.14	874	3.69 (.84)	2.98 (.81)	1.84 (.77)	1.12 (.70)	0.54 (.59)	0.1 (.58)
	dAD (NN)	2.25	874	3.72 (.94)	2.82 (.93)	1.77 (.90)	1.09 (.85)	0.45 (.78)	0.08 (.75)
SSRI	Shafer & Vovk (7)	1.05	1500	24.7	19.13	14.59	9.53	4.25	1.05
	Papadopoulos (8)	2.09	1500	10.21	8.17	6.15	3.83	2.06	1.24
	Papadopoulos (9)	1.13	1500	25.83	21.09	17.26	12.06	6.6	2.41
	Papadopoulos (10)	1.05	1500	24.57	19.69	15.33	9.87	4.7	1.21
	dAD (CV)	1.86	234	4.17 (.68)	3.15 (.63)	4.47 (.53)	1.56 (.47)	0.66 (.40)	0.23 (.21)
	dAD (NN)	2.02	234	3.98 (.89)	2.99 (.85)	2.09 (.81)	1.38 (.74)	0.63 (.67)	0.11 (.49)

Table 4: Comparison of baseline methods with proposed dynamic applicability domain (dAD) approach on benchmark datasets involving subsets of the DTC dataset, including GPCR and SSRI datasets. $\alpha_{(\delta)}$ is the median prediction region of the test set; #*calib* is number of samples in the calibration set or median number of samples for the dAD method with varying calibration sizes. Error rates represent the percent of samples with labels outside of prediction regions. Coverage values next to the dAD (CV) and dAD (NN) error rates are not reported since all results here are matched to the indices of samples where both dAD approaches were able to produce prediction regions.

DTC (GPCR; SSRI) [paired]									
		Median		Error rates per confidence level (%)					
Dataset	Approach	α_δ	#calib	75%	80%	85%	90%	95%	99%
GPCR	Shafer & Vovk (7)	1.13	1500	25.02	18.85	14.29	10.00	5.16	1.07
	Papadopoulos (8)	2.26	1500	13.81	10.83	8.14	7.71	5.11	4.56
	Papadopoulos (9)	1.25	1500	23.92	18.46	14.6	9.48	4.94	0.78
	Papadopoulos (10)	1.25	1500	24.32	18.77	14.97	10.49	5.19	1.26
	dAD (CV)	2.21	874	3.64	3.11	1.67	1.14	0.51	0.11
	dAD (NN)	2.39	874	3.62	3.04	1.69	1.06	0.42	0.12
SSRI	Shafer & Vovk (7)	1.05	1500	24.7	19.13	14.59	9.53	4.25	1.05
	Papadopoulos (8)	1.91	1500	10.02	8.4	7.06	3.98	2.27	1.94
	Papadopoulos (9)	0.84	1500	30.19	25.74	23.02	16.3	8.62	3.37
	Papadopoulos (10)	0.96	1500	24.27	19.42	15.18	9.76	4.53	1.23
	dAD (CV)	1.82	234	4.25	3.18	2.53	1.55	0.72	0.31
	dAD (NN)	1.80	234	4.53	3.32	2.68	1.67	1.01	0.17

Table 5: Error rates (%) and median α scores for dAD (NN) and dAD (CV) with applied normalisation measure as in Eq. 9

SCKBA [dAD normalised]									
		Median		Error rates per confidence level (%)					
Approach	SX	α_δ	#calib	75%	80%	85%	90%	95%	99%
dAD (NN) [norm]	S1	1.81	315	5.82 (.73)	4.32 (.74)	3.16 (.77)	3.21 (.76)	2.05 (.74)	1.21 (.63)
	S2	1.32	253	19.83 (.74)	18.17 (.7)	15.01 (.63)	13.97 (.58)	13.16 (.59)	8.2 (.6)
	S3	1.36	279	21.86 (.69)	16.42 (.71)	15.11 (.73)	12.26 (.77)	9.52 (.79)	5.76 (.73)
	S4	1.01	232	83.44 (.26)	83.12 (.39)	77.29 (.56)	76.33 (.84)	70.46 (.98)	54.62 (.87)
dAD (CV) [norm]	S1	1.52	315	6.85 (.6)	5.32 (.57)	3.97 (.54)	4.7 (.45)	3.27 (.33)	0.0 (.19)
	S2	1.24	253	20.37 (.58)	19.66 (.56)	15.89 (.48)	14.68 (.42)	11.52 (.38)	9.27 (.28)
	S3	1.16	279	23.4 (.61)	20.35 (.6)	18.06 (.56)	14.13 (.55)	10.99 (.41)	6.61 (.18)
	S4	0.95	232	83.56 (.24)	83.09 (.34)	78.69 (.51)	75.23 (.72)	72.55 (.7)	49.63 (.45)

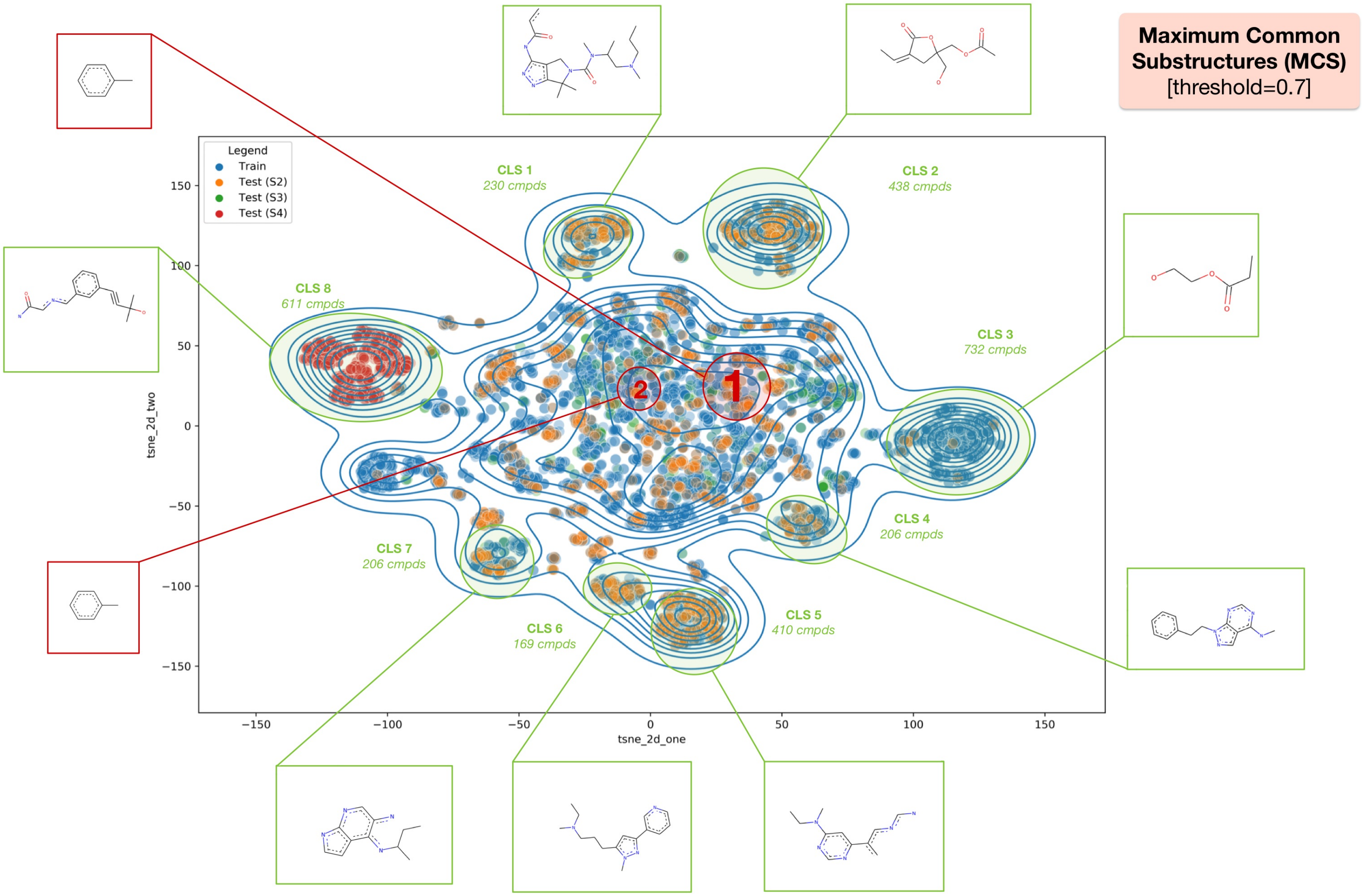

Figure 1: Results of t-SNE analysis performed over the whole chemical space of SCKBA dataset, consisting of the 7860 compounds. Compounds from the training set are shown in blue, test (S2-S4) compounds are shown in orange, green and red, respectively. Green circles show high density clusters on edges of the compound space, and for every cluster there is a maximum common substructure obtained with threshold of 0.7. Two red circles are arbitrarily assigned to the middle cloud, representing soft "clusters" with less separation between the compounds.

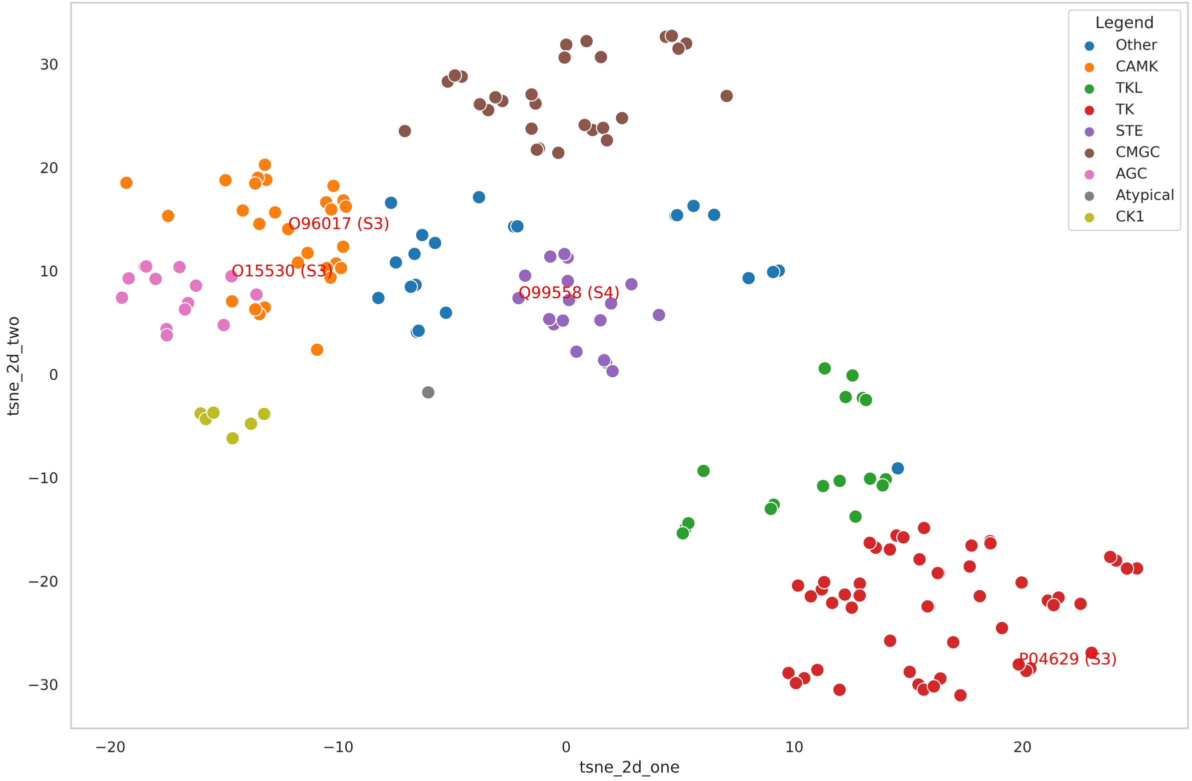

Figure 2: Results of t-SNE analysis performed over the 210 human kinases used in SCKBA dataset, with S3 and S4 targets denoted with Uniprot labels.

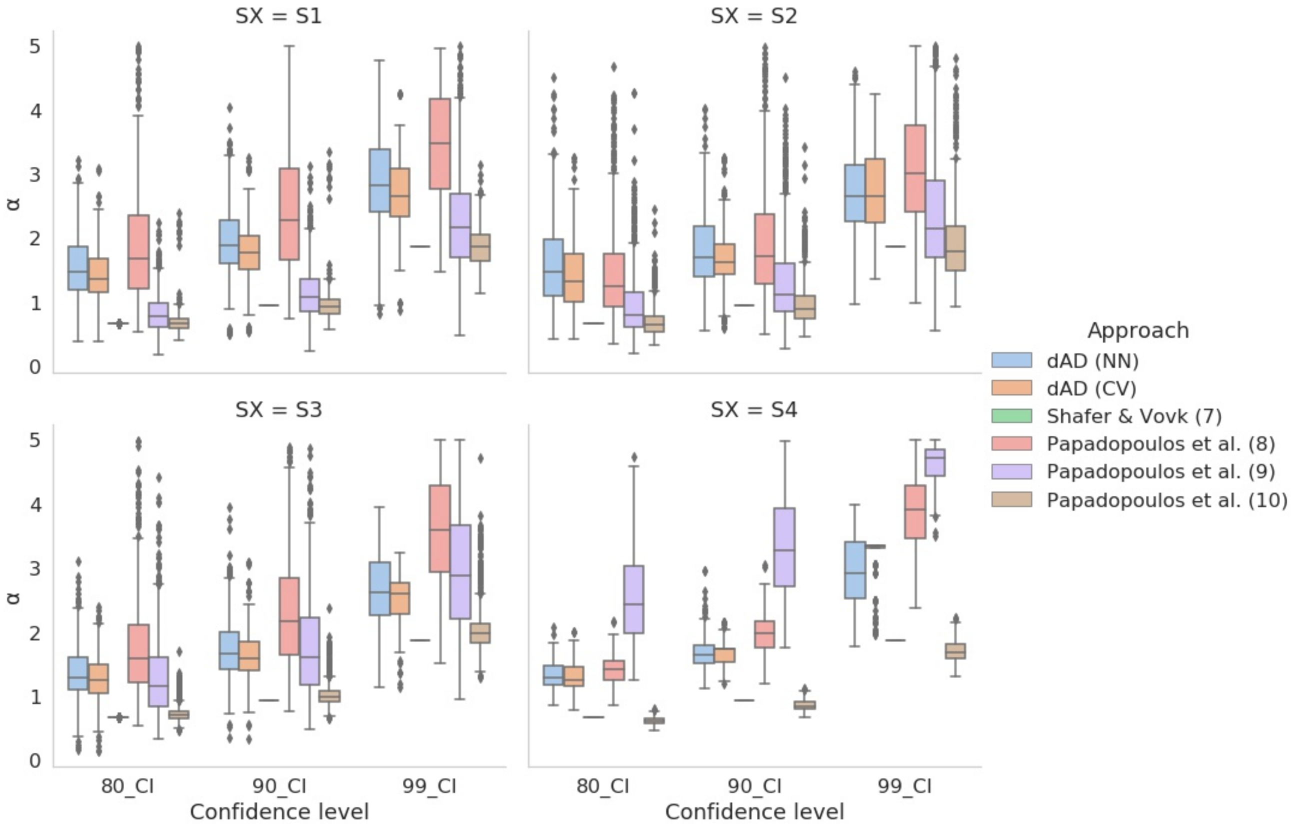

Figure 3: Comparison of original study by (Shafer and Vovk, 2008), studies with normalisation measures (Papadopoulos and Haralambous, 2010; Papadopoulos et al., 2011), and two proposed dAD variants (NN and CV) over the SCKBA dataset with four testing scenarios (S1-S4).

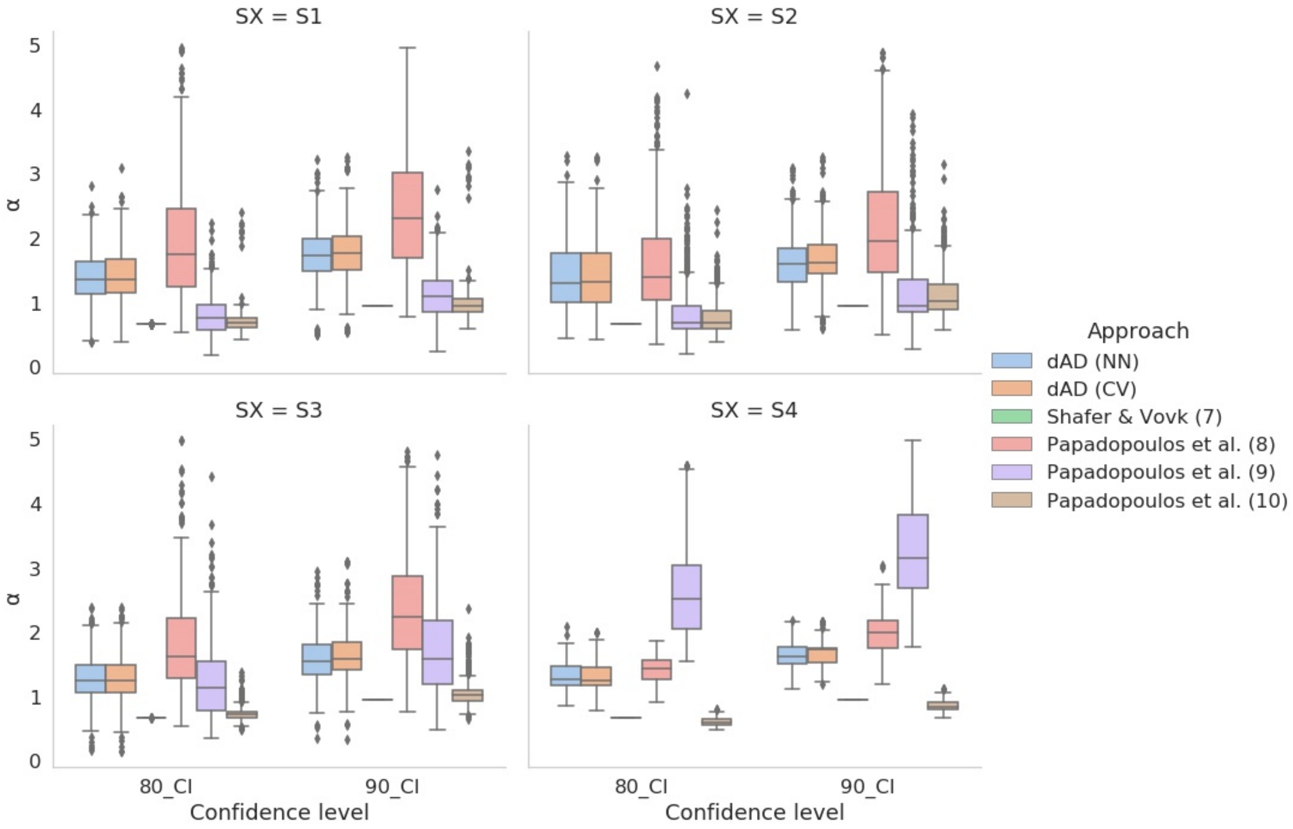

Figure 4: Comparison of original study by Shafer and Vovk (2008), studies with normalisation measures (Papadopoulos and Haralambous, 2010; Papadopoulos et al., 2011), and two proposed dAD variants (NN and CV). Results from all six of mentioned approaches are paired with indices of samples for which both dAD (NN) and dAD (CV) were able to produce prediction regions and compared over SCKBA dataset with four testing scenarios (S1-S4).

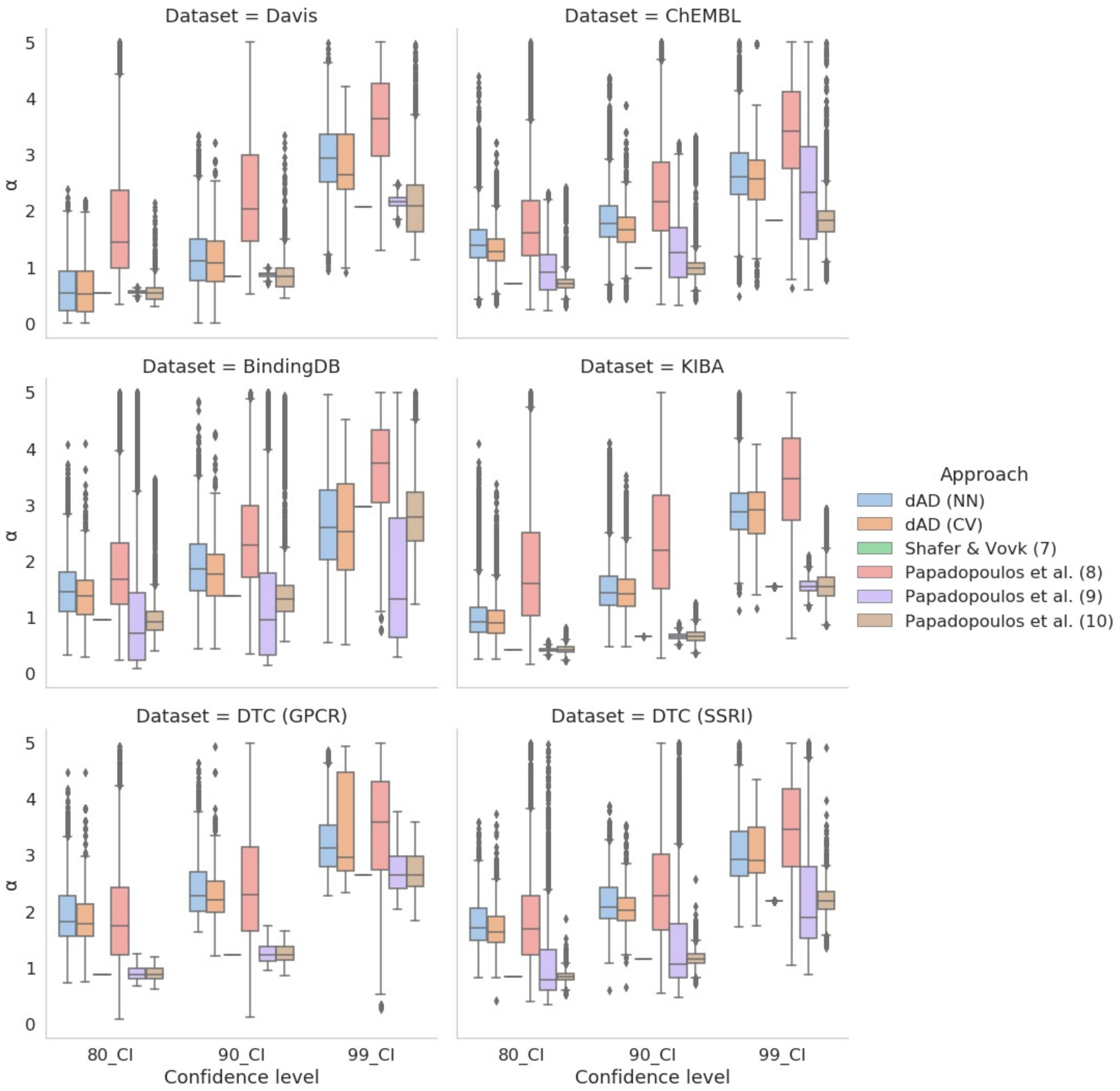

Figure 5: Comparison of original study by (Shafer and Vovk, 2008), studies with normalisation measures (Papadopoulos and Haralambous, 2010; Papadopoulos et al., 2011), and two proposed dAD variants (NN and CV) over six benchmark datasets.

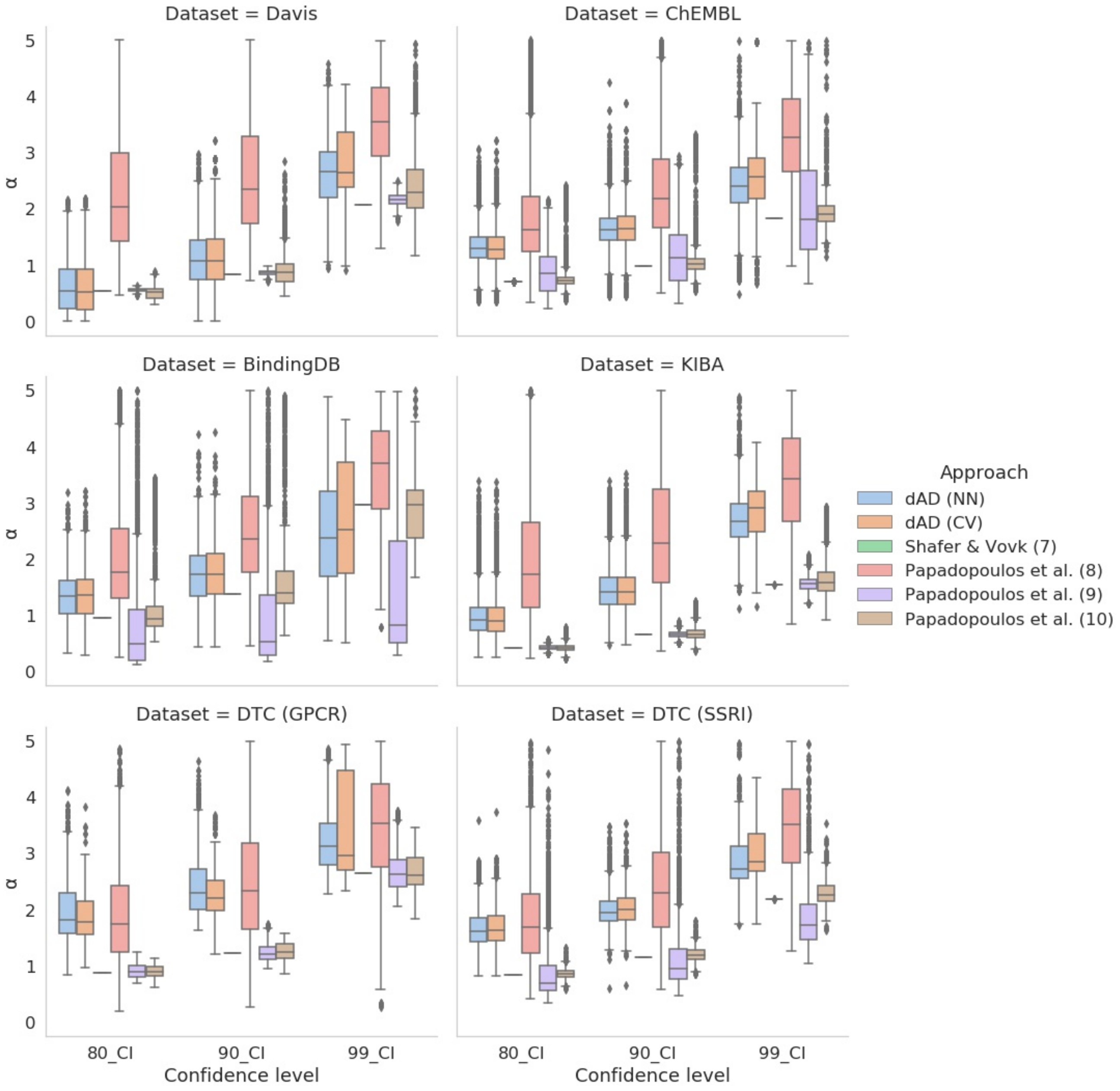

Figure 6: Comparison of original study by (Shafer and Vovk, 2008), studies with normalisation measures (Papadopoulos and Haralambous, 2010; Papadopoulos et al., 2011), and two proposed dAD variants (NN and CV). Results from all approaches over six available datasets are paired with indices of samples where for any confidence interval both dAD methods were able to produce prediction regions.

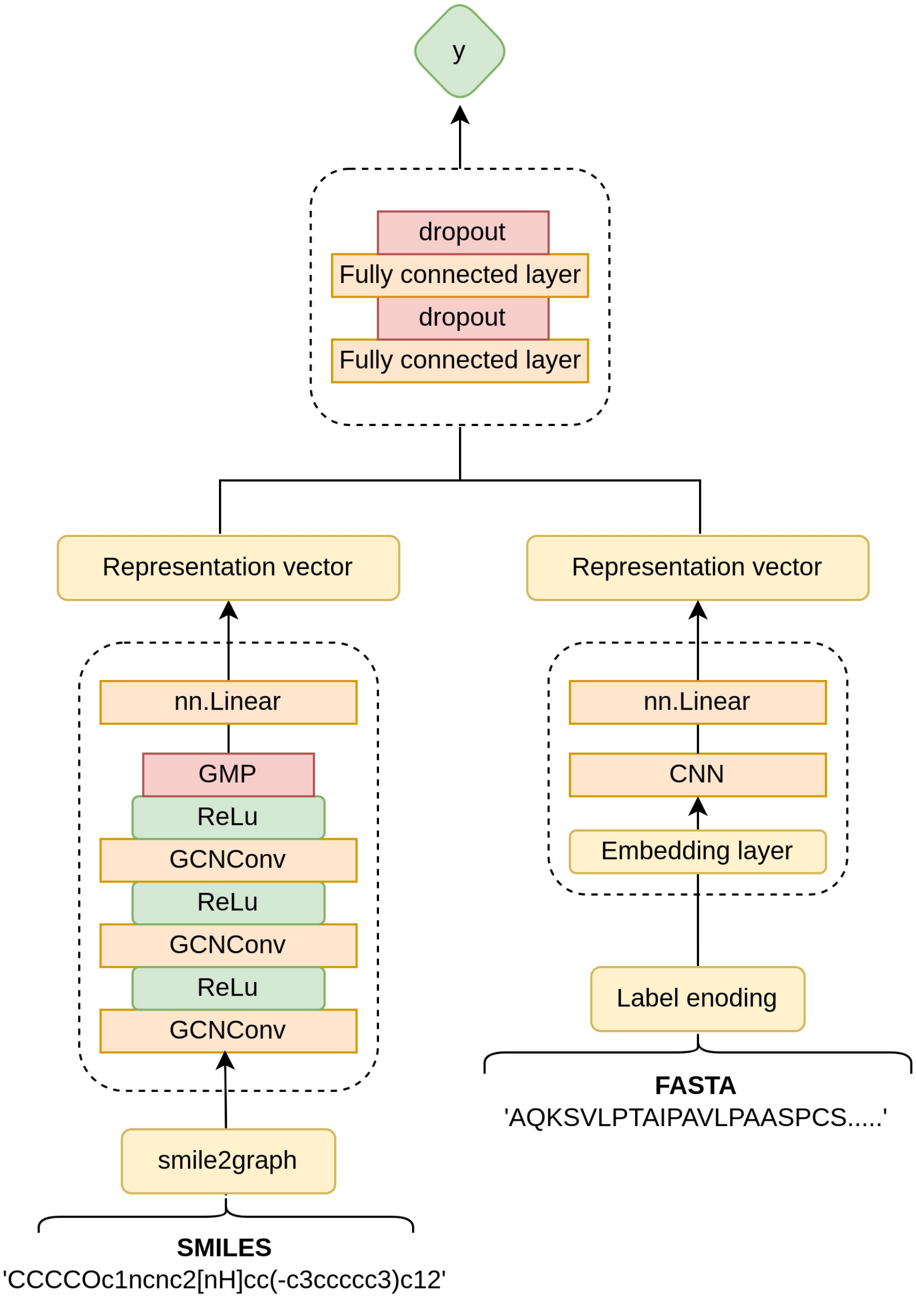

Figure 7: Schematic representation of the GCN-CNN architecture, with graph convolutional block taking SMILES as input and learning the molecular representation from graph features and convolutional block learning sequence representations from protein targets in FASTA format.

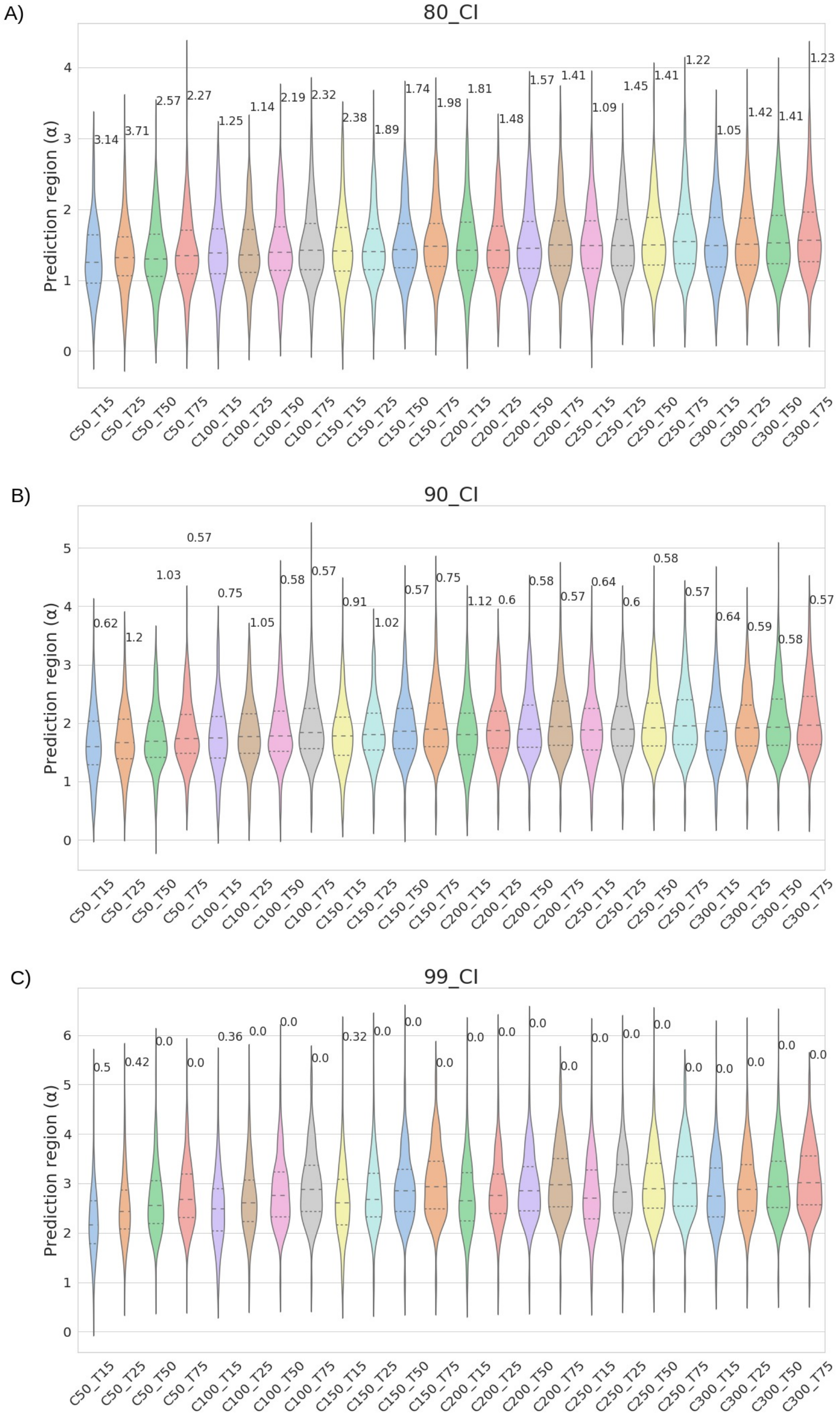

Figure 8: Figure shows the results in terms of prediction region distributions and error rates (values above each violin) for predefined confidence levels = [80%, 90%, 99%] for different number of nearest neighbours for compounds (k) and targets (q). Both hyperparameters were tuned for test (S1) scenario of SCKBA dataset.

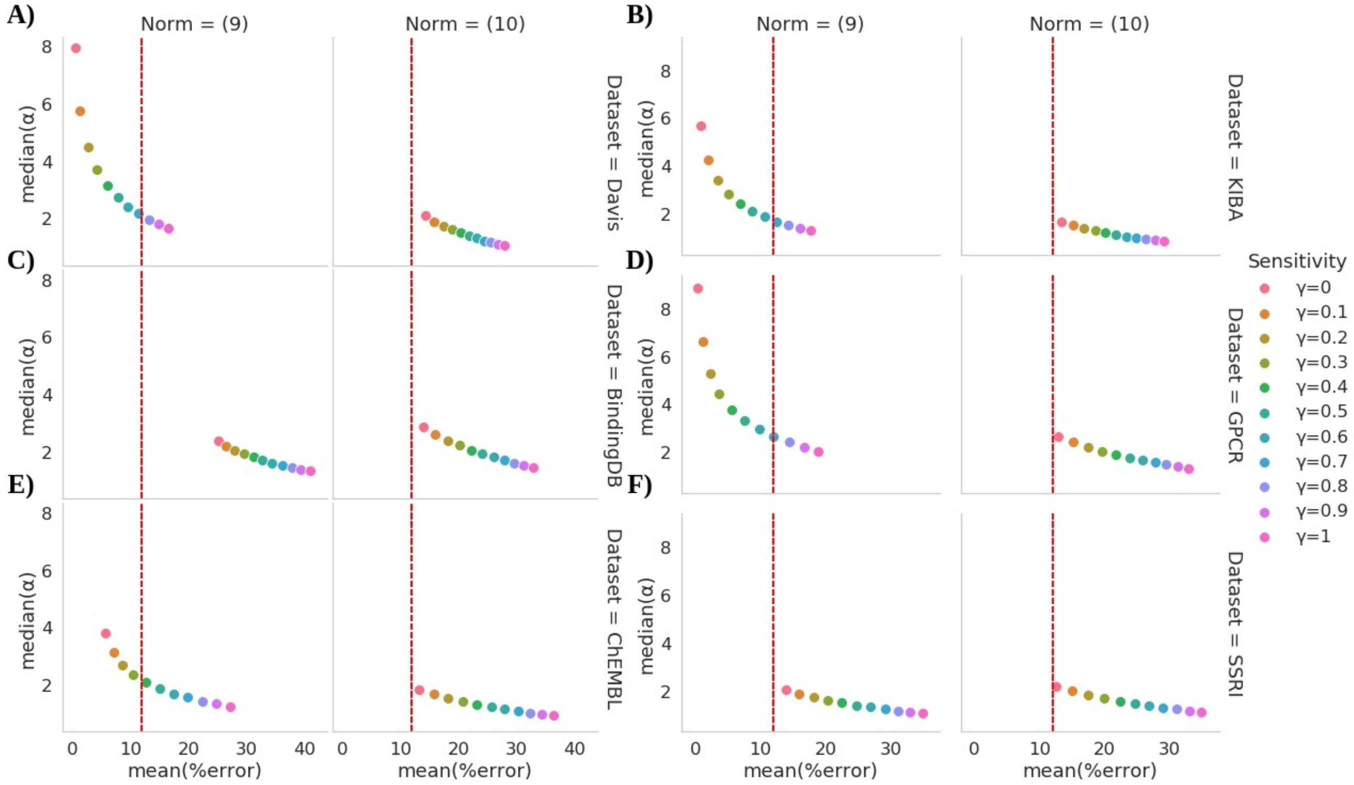

Figure 9: Figure shows the ratio between the mean error rates and median prediction region for different value of sensitivity parameter γ = [0.0, 0.1, 0.2, 0.3, 0.4, 0.5, 0.6, 0.7, 0.8, 0.9, 1] for both Papadopoulos (9) and (10) approaches, tuned for the A) Davis, B) KIBA, C) BindingDB, D) DTC (GPCR), E) ChEMBL and F) DTC (SSRI) datasets.

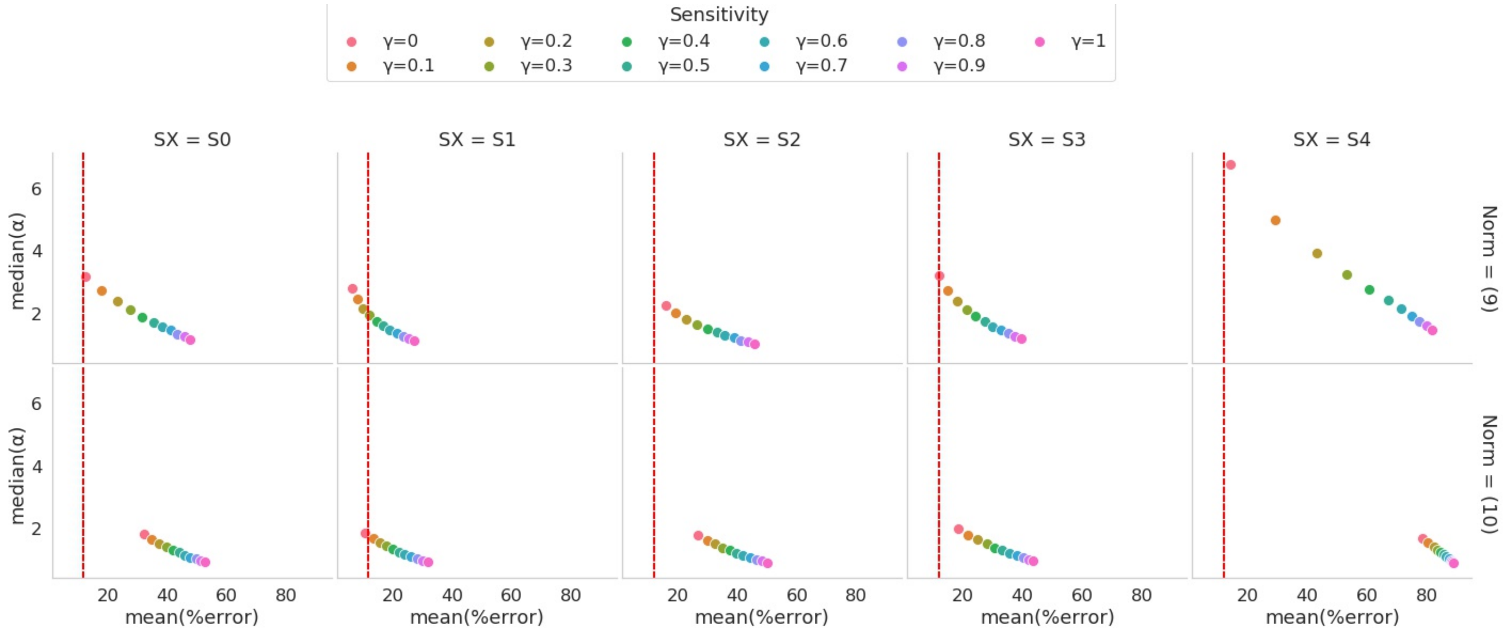

Figure 10: Figure shows the ratio between the mean error rates and median prediction region for different value of sensitivity parameter γ = [0.0, 0.1, 0.2, 0.3, 0.4, 0.5, 0.6, 0.7, 0.8, 0.9, 1] for both Papadopoulos (9) and (10) approaches, tuned for all four test scenarios of the SCKBA dataset (S1-14).

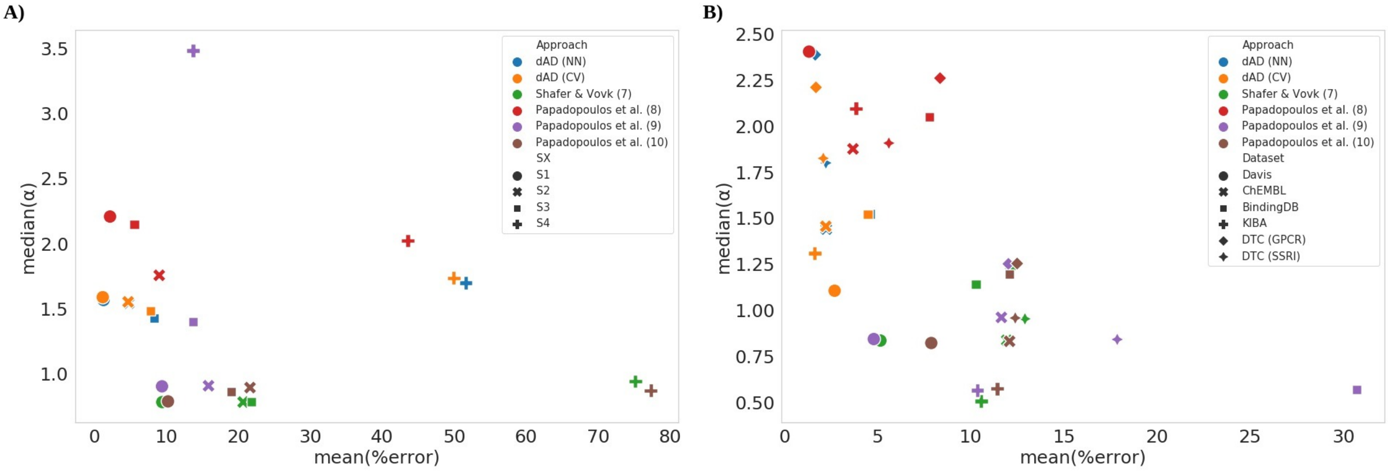

Figure 11: Comparison of the proposed dAD approach with the baseline studies by showing the relationship of mean (%) error and median nonconformity scores for A) four different testing scenarios (S1-S4) of SCKBA dataset and B) over six compound target datasets, Davis (Davis et al., 2011), KIBA (Tang et al., 2018), ChEMBL database (Gaulton et al., 2012), BindingDB (Gilson et al., 2016), DTC (GPCR) (Tang et al., 2018) and DTC (SSRI) (Tang et al., 2018) represented with different shapes. Results from all five of mentioned approaches are paired with indices of samples for which both dAD (NN) and dAD (CV) were able to produce prediction regions and compared over SCKBA dataset with four testing scenarios (S1-S4).

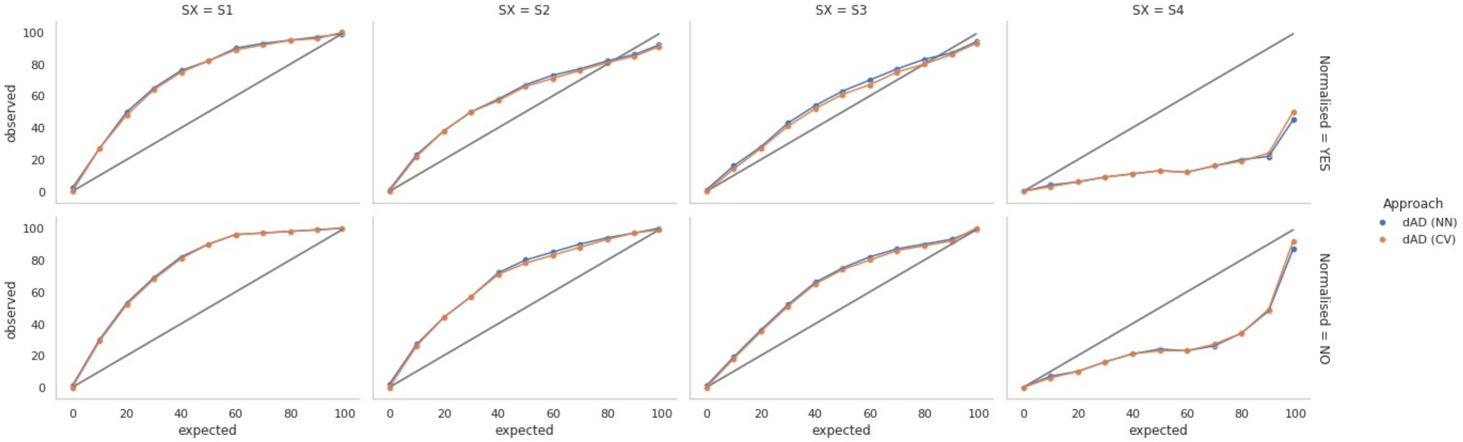

Figure 12: Linear relationship of expected and observed confidence levels for both normalised and non-normalised instances of dAD (NN), dAD (CV).

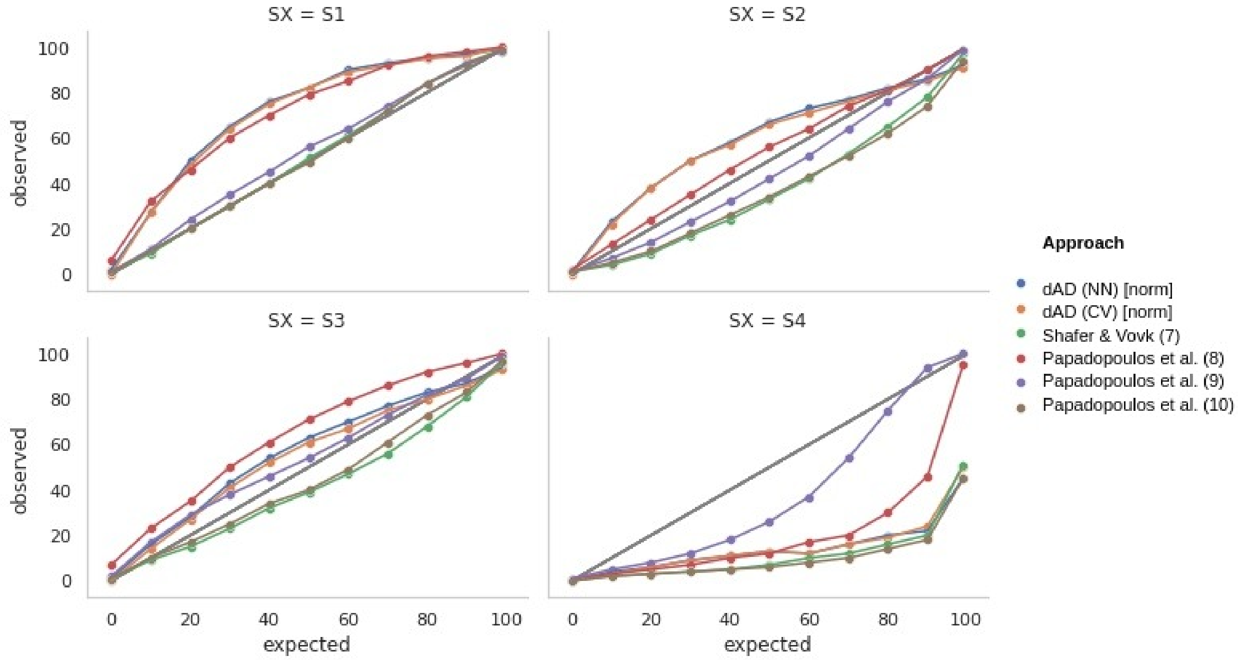

Figure 13: Comparison of the proposed dAD approach with the baseline studies over A) four different testing scenarios (S1-S4) of SCKBA dataset with paired indices across approaches and B) over six compound target datasets (Table 1) represented with different shapes, and also paired over indices to match in number of samples.

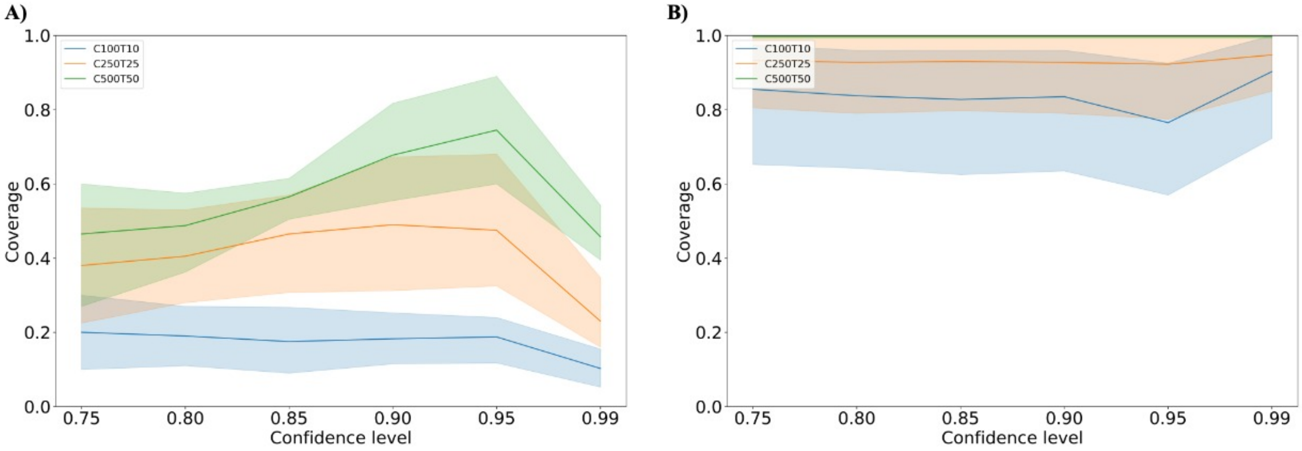

Figure 14: Demonstration of calibration size influence on the coverage of the proposed dAD approach with putative test noncoformity scores (A), and dAD coverage depending solely on calibration scores for prediction region estimation (B). Labels depict the compounds (C) and targets (T) with number of neighbours included in the conformity region of a test sample, e.g. C250T25 represents a conformity region defined by the 250 nearest neighbours in the compound space and 25 nearest neighbours in the target space of the training set.

References

M. I. Davis, J. P. Hunt, S. Herrgard, P. Ciceri, L. M. Wodicka, G. Pallares, M. Hocker, D. K. Treiber, and P. P. Zarrinkar. Comprehensive analysis of kinase inhibitor selectivity. *Nature biotechnology*, 29(11):1046–1051, 2011.

A. Gaulton, L. J. Bellis, A. P. Bento, J. Chambers, M. Davies, A. Hersey, Y. Light, S. McGlinchey, D. Michalovich, B. Al-Lazikani, et al. Chembl: a large-scale bioactivity database for drug discovery. *Nucleic acids research*, 40(D1): D1100–D1107, 2012.

M. K. Gilson, T. Liu, M. Baitaluk, G. Nicola, L. Hwang, and J. Chong. Bindingdb in 2015: a public database for medicinal chemistry, computational chemistry and systems pharmacology. *Nucleic acids research*, 44(D1): D1045–D1053, 2016.

H. Papadopoulos and H. Haralambous. Neural networks regression inductive conformal predictor and its application to total electron content prediction. In *International Conference on Artificial Neural Networks*, pages 32–41. Springer, 2010.

H. Papadopoulos, V. Vovk, and A. Gammerman. Regression conformal prediction with nearest neighbours. *Journal of Artificial Intelligence Research*, 40:815–840, 2011.

G. Shafer and V. Vovk. A tutorial on conformal prediction. *Journal of Machine Learning Research*, 9(3), 2008.

J. Tang, B. Ravikumar, Z. Alam, A. Rebane, M. Vähä-Koskela, G. Peddinti, A. J. van Adrichem, J. Wakkinen, A. Jaiswal, E. Karjalainen, et al. Drug target commons: a community effort to build a consensus knowledge base for drug-target interactions. *Cell chemical biology*, 25(2):224–229, 2018.
